# ENT1 inhibitor J4 restores cognitive function and white-matter integrity in a mouse model of tuberous sclerosis complex

**DOI:** 10.1186/s12929-026-01269-4

**Published:** 2026-06-17

**Authors:** Christine Chin-jung Hsieh, Yi-Syue Tsou, Nai-Kuei Huang, Ting-Chieh Chen, Ssu-Ju Li, Ching-Wen Chang, Wei-Xuan Lin, Szu-Yu Tung, Yijuang Chern, You-Yin Chen, Yi-Chao Lee

**Affiliations:** 1 Taipei Neuroscience Institute, Taipei Medical University, Taipei, Taiwan; 2 Biomedical Translation Research Center, Academia Sinica, Taipei, Taiwan; 3 Division of Neurosurgery, Department of Surgery, School of Medicine, College of Medicine, Taipei Medical University, Taipei, Taiwan; 4 Department of Neurosurgery, Taipei Medical University Hospital, Taipei, Taiwan; 5 National Research Institute of Chinese Medicine, Ministry of Health and Welfare, Taipei, Taiwan; 6 Ph.D. Program in Medical Neuroscience, College of Medical Science and Technology, Taipei Medical University, No. 250, Wu-Hsin St., Taipei, 11031 Taiwan; 7 Department of Biomedical Engineering, National Yang Ming Chiao Tung University, No.155, Sec.2, Linong St., Taipei, 11221 Taiwan; 8 Institute of Biomedical Sciences, Academia Sinica, Taipei, Taiwan; 9 Neuroscience Research Center, Taipei Medical University, Taipei, Taiwan; 10 International Master Program in Medical Neuroscience, College of Medical Science and Technology, Taipei Medical University, Taipei, Taiwan

**Keywords:** Tuberous sclerosis complex, White matter, Oligodendroglial maturation, CNS myelination, Equilibrative nucleoside transporter 1, Adenosine

## Abstract

**Background:**

Tuberous sclerosis complex (TSC) is a hereditary disease caused by pathogenic mutations in the *TSC1* or *TSC2* genes, leading to overactivation of mTOR signaling and dysregulation of downstream pathways. The majority of individuals with TSC develop psychiatric and neurodevelopmental comorbidities—including intellectual disability, autism spectrum disorders, anxiety and other behavioral manifestations—collectively termed TSC-associated neuropsychiatric disorders. Over the past decade, increasing evidence from clinical and preclinical studies has highlighted a significant myelination deficit in the brains of TSC patients and animal models. Although mTOR inhibition can alleviate myelination defects and improve cognition in rodent models, clinical studies using the mTOR inhibitor, everolimus, has limited cognitive benefits in TSC individuals. Adenosine signaling has been recognized as a key player in oligodendrocyte maturation and myelin formation and may represent a promising target for therapeutic intervention.

**Methods:**

In the present study, we treated *Tsc2*^+*/–*^ mice with J4, an equilibrative nucleoside transporter 1 inhibitor that has been demonstrated to increase adenosine level, and used diffusion MRI and relevant imaging modalities to assess its therapeutical effects on behavioral deficits and white matter defects in *Tsc2*^+*/–*^ mice. We also analyzed the myelin ultrastructural changes using transmission electron microscopy and examined the expression oligodendrocytes- and myelination-associated proteins after the treatment.

**Results:**

J4 treatment improved both cognitive deficits and anxiety-like behavior in *Tsc2*^+*/–*^ mice, and ameliorated white matter abnormalities through enhancing myelin sheath integrity. We also demonstrated that J4 mitigates gray matter cytoskeletal disorganization, along with increased expression of key mature oligodendrocyte- and myelin-associated proteins. Furthermore, J4 significantly reduced the aberrant overexpression of pS6 and cFos, both of which are elevated in *Tsc2*^+*/–*^ mice as a result of hyperactivation of mTOR and heightened neuronal activity.

**Conclusion:**

Altogether, these findings indicate that J4 modulates oligodendroglial lineage populations, enhances myelination, and improves neural connectivity by regulating neuronal hyperactivity. Our results suggest that J4 is a strong therapeutic candidate for addressing the neuropsychiatric manifestations of TSC.

**Supplementary Information:**

The online version contains supplementary material available at 10.1186/s12929-026-01269-4.

## Introduction

Tuberous sclerosis complex (TSC) is an autosomal dominant hereditary disease, which is characterized by the formation of non-cancerous tumors known as hamartomas in various organ systems, notably affecting the brain, skin, kidneys, and eyes [1, 2]. Mutations in the TSC1 or TSC2 genes are known to cause over-activation of the downstream protein mTOR [1, 3], leading to uncontrolled cell growth and the formation hamartomas, as well as neurologic lesions, namely cortical tubers, subependymal nodules (SENs), and subependymal giant cell astrocytoma (SEGA). Despite the notable hamartoma and lesions, seizures and cognitive impairment are the primary source of patient and caretaker burden. Nearly 90% of TSC patients had a history of epilepsy, of which up to 60% of them developed refractory seizures [4, 5]. In addition, TSC is also associated with a wide range of cognitive, behavioral and psychiatric manifestations, which is under an umbrella term: TSC-associated neuropsychiatric disorders (TAND) [6].

TAND manifestations can be further divided into six categories: psychiatric, intellectual, behavioral, academic, neuropsychological, and psychosocial levels [6]. It is estimated that half of individuals with TSC present with varying degrees of intellectual disability [7]. Approximately 25%-50% of children with TSC develop autism spectrum disorder (ASD) [6, 8, 9]. At the behavioral level, the most common symptoms include overactivity (45%), sleep difficulties (43.9%), and anxiety (33.3%). Around 90% of individuals with TSC will show at least one of these TAND manifestations during their lifetime [10].

Despite the high incidence of TAND in TSC, a significant proportion of patients are under-diagnosed and lack effective pharmacological treatments for these neuropsychiatric manifestations [11]. Despite its efficiency in the rodent models [12–14], the mTOR inhibitor rapamycin could not ameliorate the neurocognition and autistic behaviors [15–17]. The existing treatments for TAND rely largely on non-drug interventions, such as physical, occupational, and/or speech therapies for autism-related symptoms. Importantly, according to a report from a patient-focused drug development meeting with the US FDA, many individuals with TSC emphasized that an ideal therapeutic should directly target TAND symptoms rather than only seizures or tumor growth [18, 19]. Together, these perspectives underscore the urgent need for novel pharmacological strategies that act through alternative mechanisms to alleviate TSC-related cognitive and psychological deficits.

Several studies have reported it is likely a result of a complex interplay of the wide range of identified risk factors involves gene mutations and aberrant signaling pathways within the disease [20, 21]. It is widely believed that the disruptions in the brain structure and function resulting from dysregulated mTOR signaling contribute to the development of TAND in TSC. Abnormalities in synaptic plasticity, cytoskeletal organization, and white matter (WM) integrity have all been implicated in these neurobehavioral phenotypes [12, 22–27]. Notably, diffusion tensor imaging (DTI) studies in TSC patients have consistently revealed microstructural WM abnormalities, despite often normal-appearing WM on conventional MRI, including reduced fractional anisotropy (FA) and increased radial diffusivity (RD) in major tracts such as the corpus callosum [28–30]. Animal studies also confirmed subtle myelin and axonal defects in TSC [25, 31]. These findings support the notion that structural brain network disruptions originating from the myelination impairments may underlie TAND.

Central nervous system (CNS) myelination is an essential and highly orchestrated process involving the proliferation, differentiation, and maturation of a type of glial cells called oligodendrocytes (OLs) [32]. During the myelination process, mature OLs extend their cellular processes to wrap around the axons with multiple layers, forming compact myelin sheaths, enabling rapid and efficient electrical conduction, as well as providing metabolic support [33]. Proper myelination is vital for normal brain development, neuronal communication, and cognitive function. Conversely, impaired myelination can disrupt connectivity between brain regions, affecting information processing and transmission of signals, potentially contributing to neuropsychiatric symptoms [34, 35].

Multiple studies have reported reductions in both myelin content and the number of OLs in the brains of individuals with TSC [22, 36–38]. These deficits not only occur within cortical tubers but also throughout widespread WM regions. Cellular and animal studies further revealed that the hyperactive mTOR signaling adversely influences OL development and maturation [25, 31, 39–41], which is considered as the pivotal underlying mechanism responsible for WM malformation seen in TSC individuals. Taken together, these findings suggest myelination enhancement as a potential therapeutic strategy for improving TAND from birth to adulthood.

Adenosine is a well-characterized, endogenous neuromodulator in the CNS that influences various cellular processes including myelination. Oligodendrocyte precursor cells (OPCs) express all adenosine receptor subtypes (A_1_, A_2A_, A_2B_, and A_3_), which have distinct roles in regulating the differentiation and maturation of OLs [42]. Activation of A_1_ receptors by adenosine promotes OL maturation and inhibits proliferation, facilitating OPCs to transform into mature myelinating OLs. Conversely, activation of A_2A_ receptors inhibits OL maturation, demonstrating a balance is precisely controlled through adenosine receptor signaling in mediating myelination dynamics. The equilibrative nucleoside transporter 1 (ENT1) regulates extracellular adenosine levels by mediating its bidirectional transport of adenosine across the cell membrane [43, 44]. Inhibition of ENT1 leads to increased extracellular adenosine, enhancing A_1_ receptor-mediated signals that favor OL differentiation and maturation. Therefore, targeting ENT1 with inhibitors to raise extracellular adenosine level represents a promising strategy to modulate OL development and myelination, with potential therapeutic implications for neurological disorders involving myelin deficits.

J4, an ENT1 inhibitor, has been demonstrated to enhance cognitive function in various preclinical studies, particularly in models of Alzheimer’s disease (AD). For example, oral administration of J4 effectively ameliorates the cognitive decline and the accumulation of misfolded disease-causing proteins (i.e., amyloid plaque and hyperphosphorylated tau) in two distinct mouse models of AD, APP/PS1 and THY-Tau22, respectively [45–47]. J4 was demonstrated to rescue the mitochondrial dysfunction, and ameliorate the cellular energy imbalance by modulating the nucleoside metabolism and homeostasis [45, 48]. In this present study, we aimed to elucidate the effects of J4 treatment on strengthening the cognitive functioning in *Tsc2*^+*/–*^ mice via promoting the OL differentiation/maturation and myelination, while also emphasizing the application of advanced diffusion imaging, such as DTI and diffusion kurtosis imaging (DKI), in detecting microstructural WM pathology and monitoring J4 treatment responses.

## Materials and methods

### Animals

Animals used in this study were treated in accordance with guidelines of the University Committee on the Care and Use of Experimental Animals of Taipei Medical University (Taipei, Taiwan). Mice were housed in an air-conditioned vivarium with free access to food and water and a 12/12-h light/dark cycle. Only male animals (6-week-old to 12-week-old) were used and female mice were not used due to hormonal cycle variability, which may affect the behavioral tests that were carried out. The *Tsc2*^+/–^ knockout mouse model (B6;129S4-Tsc2tm1Djk/J) was purchased from Jackson laboratory (Bar Harbor, ME, USA). *Tsc2*^+*/−*^ mice and their wild-type (WT) littermates were generated from crossing *Tsc2*^+*/−*^ mice with WT. WT mice were used as littermate controls in all experiments, ensuring a matched genetic background and minimizing confounding effects due to strain differences.

### Drug treatments

J4 treatment was given ad libitum in the drinking water at the concentration of 0.06 mg/ml. J4 was dissolved in the drinking water containing 1% HPβCD, which served as the vehicle. The total treatment time was 10 weeks. The average daily intake for a laboratory mouse was estimated to be 0.17 ml/g/day [49]. Thus, the dose of 0.06 mg/ml J4 in drinking water would approximately give an overall dose of 10.2 mg/kg/day for the mice in this study. Animals were randomly assigned to experimental groups.

### Novel object recognition test

Before each behavioral test session, mice were habituated in the behavior room for 30 min to 1 h. The novel object recognition (NOR) task consisted of three days: habituation, familiarization, and testing. During habituation, mice explore an empty arena (58 × 58 × 35 cm) for 10 min. In familiarization, two identical objects (A + A) are placed in the arena for 10 min. In testing, one familiar object (A) and one novel object (B) are presented for 10 min. Exploration time for each object is recorded, and the discrimination index (DI) is calculated as ((B − A)/(B + A))((B—A) / (B + A))((B − A)/(B + A)).

The open-field test (OFT) was carried out using the first day of the NOR test. Mice were allowed to explore the arena freely without any objects for 10 min. A center region with dimensions of 29 × 29 cm was selected to observe how much time the animal stayed in the region. Motor tracks of all behavioral tests were video-recorded and analyzed by an open-source Matlab program, OptiMouse [16]. Behavioral testing was performed by investigators aware of group allocation; all behavioral data analyses were conducted by investigators blinded to group identity. In addition, all imaging and histological analyses were performed by investigators blinded to group allocation.

### Brain slice preparations and immunostaining

Mice underwent transcardial perfusion and fixation using 4% paraformaldehyde and then decapitated. After extracted from the skull, the brains underwent post-fixation with 4% paraformaldehyde at 4 ℃ overnight. Fixed brains were dehydrated in 30% sucrose in 0.5 M PB for at least 4 days prior to the frozen sectioning. Sections with 30–50 μm-thickness were obtained using Thermo Fisher HM430 Microtome (Thermo Fisher Scientific, Waltham, MA, USA).

For immunofluorescence staining, brain slices were incubated with desired antibodies for overnight at 4℃. The following primary antibodies were used in this study: pS6 (Ser235/236 #4858, Cell Signaling, MA, USA), cFos (SC-271243, Santa Cruz Biotechnology, CA, USA), SMI-31 (NF) (NE1022, Merck, Darmstadt, Germany), NG2 (AB5320, Millipore/Sigma, CA, USA), APC, ab16794, Abcam, Cambridge, UK), MBP (808,401, BioLegend, CA, USA), PLP (ab105784, Abcam), CNPase (MAB326, Merck), and Ctip2 (ab18465, Abcam). After PBS washes, the slices were incubated with the corresponding Alexa Fluor dye-tagged secondary antibodies and cell nuclei were counterstained with Hoechst 33,258 (Sigma-Aldrich, Burlington, MA, USA) at room temperature for 1.5 h. After 3 times of PBS washes, brain slices were mounted onto the slide(s) and mounted with anti-fading mounting medium (Vector Laboratories, Burlingame, CA, USA). Images were acquired by Leica Stellaris 8 confocal fluorescent microscope (Leica Biosystems, Wetzlar, Hesse, Germany).

For Luxol Fast Blue (LFB)-Cresyl Violet staining, the brain slices were first mounted onto the slides and LFB stain kit (ab150675, Abcam) was used according to the manufacturer’s manual. Briefly, the slides with the brain sections were placed in the LFB solution for 2 h at 60 °C. The slides were rinsed with MQ water for 5 min, followed by briefly soaked with lithium carbonate solution. Then the slides were subsequently washed with 70% alcohol for about 1 min and followed by rinsing with MQ water for 1 min twice. Dehydration in graded alcohols (i.e., 50%, 75%, and 95% alcohol). Finally, the slides were cleared in xylene and mounted with mounting medium. Images were acquired by TissueGnostics and visualized with TissueFAXS & HistoFAXS (TissueGnostics GmbH).

### Transmission electron microscopy and image analysis

Mice underwent transcardial perfusion with perfusion fixative buffer and decapitated. The brains then underwent post-fixation with 2% PFA/2.5% gluataraldehyde in 0.1 M Cacodylate, pH 7.4 at 4 °C overnight. The fixed brains were then washed with 0.1 M Cacodylate/7% sucrose and post-fixed with 1% OsO4 for 1–2 h. Next, the brains were washed again with 0.1 M Cacodylate/7% sucrose, followed by dehydration steps, which was incubation of ethanol with subsequent concentrations, 70%, 80%, 90%, 95%, and 100% for 15–45 min. The brain tissues were then placed in Propylenoxide for 10 min 3 times and Propylenoxide/EPON (with a 1:1 ratio) for overnight. Embedding and hardening at 62 °C for 3 days, and then followed by sectioning. Electron micrographs were obtained using HT7700 (HITACHI, Tokyo, Japan). For g-ratio and myelin thickness calculations, Fiji/ImageJ (NIH, Bethesda, MD, USA; https://imagej.net/Fiji) [50] was used to first define the diameters of axons and myelin sheath surface area. The formula is as follows: G = d/D, where d is the diameter length of the axon and D is the diameter length of the axon with myelin sheath. The myelin thickness is determined by the surface area of the axon including myelin minus the surface area of the axon.

### Confocal images quantitative analysis

For general quantification, for each group, 3 random areas of the desired brain region were selected from different animals (N = 3–5) and analyzed. The area percentage of immunoreactivity of each antibody was determined by the “Measure” function of the Fiji/ImageJ after “Background subtraction” to correct the uneven illuminated background. The values presented were normalized to the control or WT mice. The number of cells was counted by the “Analyze Particles” function of the Fiji software and validated by manual counting by a different experimenter.

For pS6-positive cells quantification, images were first processed in Fiji/ImageJ, and a fixed intensity threshold was applied to all groups to identify p-S6-positive cells. The threshold was defined based on signal intensity above basal levels and was kept constant across all experimental conditions to ensure comparability. Cells exceeding this threshold within the defined ROI were counted as p-S6-positive.

Coherency analysis was performed as previously described [51]. Briefly, MBP-immunostained brain sections were analyzed to assess the directional organization of myelinated fibers using the OrientationJ plugin in Fiji/ImageJ. For each animal, three regions-of-interest (ROIs; 200 μm × 200 μm) were randomly selected within the stratum radiatum (sr) of the hippocampal CA1 region. Prior to analysis, images were subjected to background subtraction to remove non-directional fluorescence signal. Coherency values within each ROI were then computed using the “OrientationJ Measure” function, which quantifies the degree of local alignment of myelinated fibers. ROIs used for analysis are indicated in Fig. 5F.

For morphological analysis of CNPase-positive OLs, cortical layers in the retrosplenial (RSP), parietal, and somatosensory (SS) cortices were first defined by mapping to the Allen Brain Atlas (Supplementary Fig. 1A–C). Then, only cells with clearly identifiable cell bodies and sufficiently well-defined processes to allow reliable tracing were included for morphological analysis (Supplementary Fig. 1D–G). CNPase-positive OLs were imaged, converted to 8-bit images, and processed into binary images. Morphological reconstruction was then performed using the Simple Neurite Tracer (SNT) plugin [52] in Fiji/ImageJ to trace cellular processes and generate schematic representations. For quantification, 20 OLs per group were reconstructed and analyzed, with cells sampled from 3–4 animals per group.

### MRI acquisition and diffusion MR imaging data analysis

MRI acquisition and analysis were obtained according to our previous study [53]. Briefly, we scanned the mice on a 7-Tesla small-animal system (Biospec 70/30 USR, Bruker, Ettlingen, Germany) with a planar surface coil (T7399V3; Bruker) placed over the head for signal reception. Anesthesia was maintained at 3% isoflurane in 20% O_2_, 75% N_2_, and 5% CO_2_ throughout the scanning. Diffusion-weighted images were obtained using a spin-echo EPI sequence (DtiEpi) with TR/TE = 3,750/31/2 ms, matrix 128 × 128, FOV = 20 × 20 mm^2^, and voxel size 0.08 × 0.08 × 0.4 mm^3^. Five b0 volumes plus 90 diffusion-weighted volumes were collected across three shells (b = 500, 1,000, 2,000 s/mm^2^, 30 directions per shell, three averages each).

MR images from DTI was processed and evaluated using DSI Studio (http://dsi-studio.labsolver.org/) [54]. Tract-based and atlas-based ROI analysis were conducted to compare group differences in DTI parameters (FA, RD). The “Color FA” and “Region FA” data are extracted from DSI Studo and are used to illustrate the directional orientation of white matter fiber tracts (green: anterior-to-posterior; red: left-to-right; blue: dorsal-to-ventral), and the FA values, respectively. After registration to the Allen Mouse Brain Atlas (FLIRT, FSL), ROI-based metrics were extracted for DKI metrics, including mean kurtosis (MK), and radial kurtosis (RK). These metrics were computed with in-house MATLAB scripts (MATLAB 2021a; MathWorks, MA, USA). White matter tract integrity (WMTI) metrics and axonal water fraction (AWF) was calculated via weighted least-squares fitting in DIPY (version 1.11.0) [55].

### Statistical analysis

NOR discrimination indices and time spent in the center arena for the OFT were compared among the groups using one-way ANOVA and *post-hoc* analysis of Bonferroni test to determine the statistical significance. A *p*-value less than 0.05 (*p* < 0.05) indicates a statistically significant result. For immunostaining image analyses, the average number of pS6-, cFos-, APC-, NG2-positive cells, and the immunoreactivity of MBP, PLP1, CNPase were assessed using one-way ANOVA with Bonferroni test as post hoc analysis for multiple comparisons. The datasets were tested and confirmed for the normality using D'Agostino & Pearson, Anderson–Darling, and Kolmogorov–Smirnov tests. For diffusion MRI metrics among the three groups, Kruskal–Wallis with Dunn’s test as the post hoc analysis for multiple comparisons was used. For CNPase-positive OL morphological analysis, comparisons between groups were carried out using Fisher’s exact test. All statistical analyses mentioned above were performed using Prism version 10 (GraphPad Software, Inc., San Diego, CA, USA).

## Results

### *J4 ameliorated cognitive deficits and anxiety-like behavior in Tsc2*^+*/–*^* mice*

**Fig. 1:**
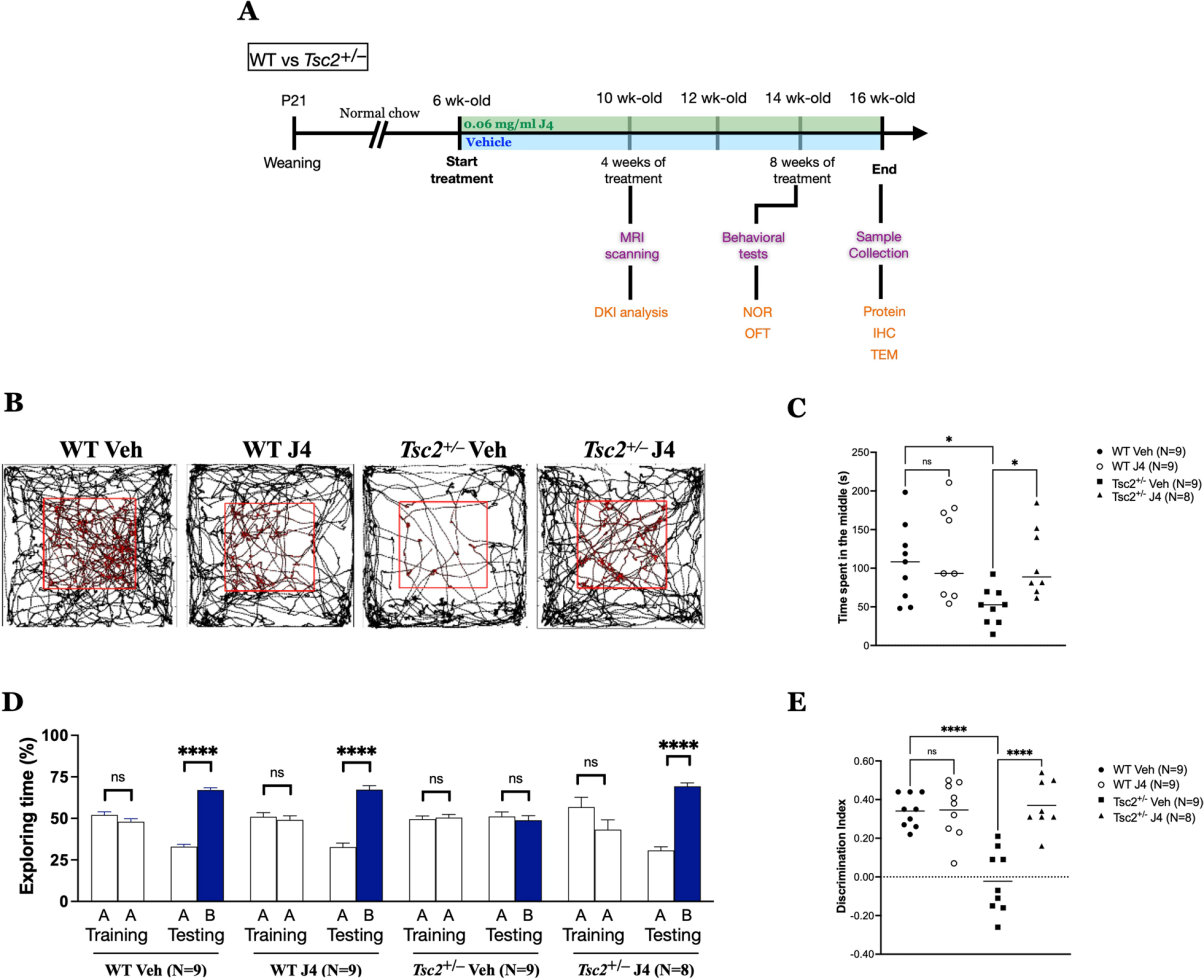
*J4 ameliorated cognitive deficits and anxiety-like behavior in Tsc2*^+*/–*^* mice.* **A** Schematic timeline of the experimental design. **B** Representative tracings during the OFT among wild-type (WT) and *Tsc2*^+*/–*^ mice treated with vehicle (Veh) or J4: WT Veh, WT J4, *Tsc2*^+*/–*^ Veh, and *Tsc2*^+*/–*^ J4 groups. **C** Quantification of the time spent in the center zone of the open-field arena. **D** Percentage of time spent exploring object A (white bar) and object B (blue bar) during the training and testing phases of the NOR task in the four groups. **E** Discrimination index of the NOR task for each group. Statistical analyses were performed using paired *t*-tests and one-way ANOVA followed by Bonferroni post hoc correction for multiple comparisons. Data are shown as mean ± SEM.*p < 0.05, **p < 0.01, ***p < 0.001, ****p < 0.0001, *n.s.* not significant, *DKI* diffusion kurtosis imaging, *NOR* novel object recognition, *OFT* open-field test, *IHC* immunohistochemistry, *TEM* transmission electron microscopy

The *Tsc2*^+*/–*^ mice employed in this study display cognitive dysfunction and ASD-like features in the absence of brain hamartomas and spontaneous seizures, making them well suited for investigating neurobehavioral deficits solely driven by intrinsic *Tsc2* deficiency [12, 51, 53]. To test whether J4 is effective in treating cognitive deficits in *Tsc2*^+*/–*^ mice, we treated the WT littermates and *Tsc2*^+*/–*^ mice with a long-term diet containing a normal chow with J4 in drinking water for 10 weeks (WT J4 and *Tsc2*^+*/–*^ J4), and vehicle in drinking water to serve as controls (WT Veh and *Tsc2*^+*/–*^ Veh). An experimental procedural diagram was shown in Fig. 1A. We assessed the anxiety-like behavior by analyzing the time spent exploring in the center region for each group using the open-field test. Based on the tracking data and quantitative analysis, *Tsc2*^+*/–*^ Veh spent significantly less time exploring the center region of the arena compared with the WT Veh and *Tsc2*^+*/–*^ J4, while WT J4 showed no differences compared to the WT Veh (Fig. 1B, C; F (3, 31) = 4.082, *p* = 0.0149, one-way ANOVA; Bonferroni post hoc comparisons: WT Veh vs. *Tsc2*^+*/–*^ Veh, *p* = 0.0475; WT Veh vs. WT J4, *p* > 0.9999; *Tsc2*^+*/–*^ J4 vs. *Tsc2*^+*/–*^ Veh, *p* = 0.0484).

Moreover, during the NOR test, *Tsc2*^+*/–*^ Veh mice were unable to distinguish the familiar object A and the novel object B, and spent less time exploring the novel object. On the other hand, WT Veh and WT J4 mice spent significantly longer exploring time for the novel object B (Fig. 1D). In *Tsc2*^+*/–*^ J4 mice, the exploring time was increased for the novel object. The DI was calculated for each group (Fig. 1E**).** The DI for *Tsc2*^+*/–*^ Veh group was significantly lower than the other 3 groups, indicating that *Tsc2*^+*/–*^ mice exhibited deficits in learning and memory function and the treatment of J4 was able to improve the deficits (Fig. 1E; F(3, 31) = 17.59, *p* < 0.0001, one-way ANOVA; Bonferroni post hoc test, *p* < 0.0001).

### *Normal-appearing white matter (NAWM) of Tsc2*^+*/–*^* mice*

**Fig. 2:**
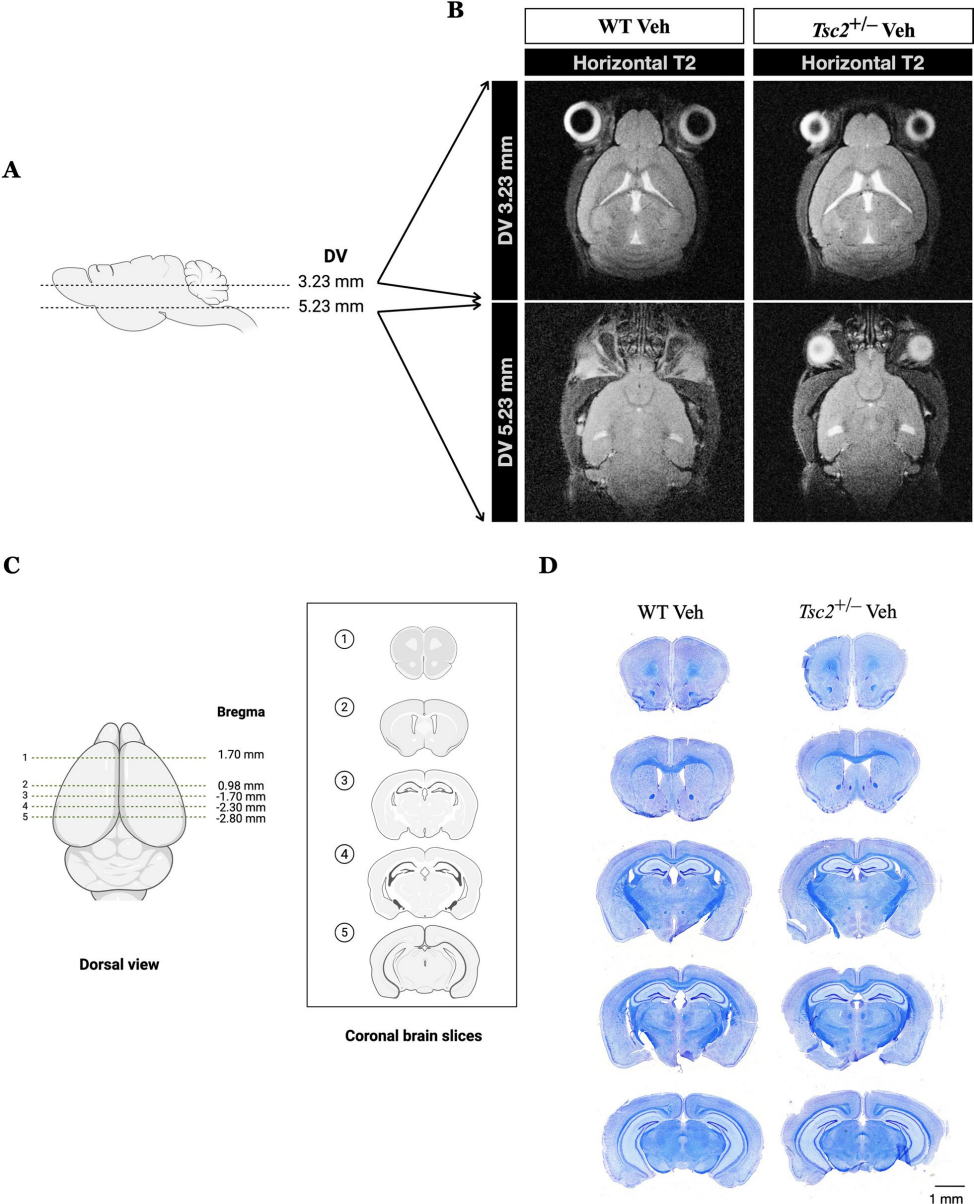
*Normal-appearing white matter (NAWM) of Tsc2*^+*/–*^* mice*. **A** Schematic diagram showing the sagittal brain section at the indicated dorsal–ventral positions. **B** Representative horizontal T2-weighted MRI images for each of the indicated groups are shown. **C** Schematic illustration showing the dorsal view of the mouse brain with the corresponding coronal slice levels relative to bregma (left). The number diagrams represent the coronal brain sections at the indicated anterior–posterior coordinates (right). **D** Representative Luxol Fast Blue-stained coronal brain sections from wild-type (WT) and *Tsc2*^+*/–*^ mice treated with vehicle (Veh): WT Veh and *Tsc2*^+*/–*^ Veh. Images are shown at matched anterior–posterior positions. Scale bar: 1 mm

Clinical MRI scans from individuals with TSC typically show NAWM, suggesting that conventional MRI may not detect subtle white matter abnormalities [56]. Consistently, we found that *Tsc2*^+*/–*^ mice also exhibit NAWM on T2-weighted MRI (Fig. 2A, B). To further assess the overall myelination pattern at the histological level, we employed LFB staining on serial brain sections from WT and *Tsc2*^+*/–*^ mice (Fig. 2C, D). The staining revealed that no apparent differences in the WM architecture between the two groups.

### *J4 ameliorated diffusion MRI abnormalities in Tsc2*^+*/–*^* mice*

**Fig. 3:**
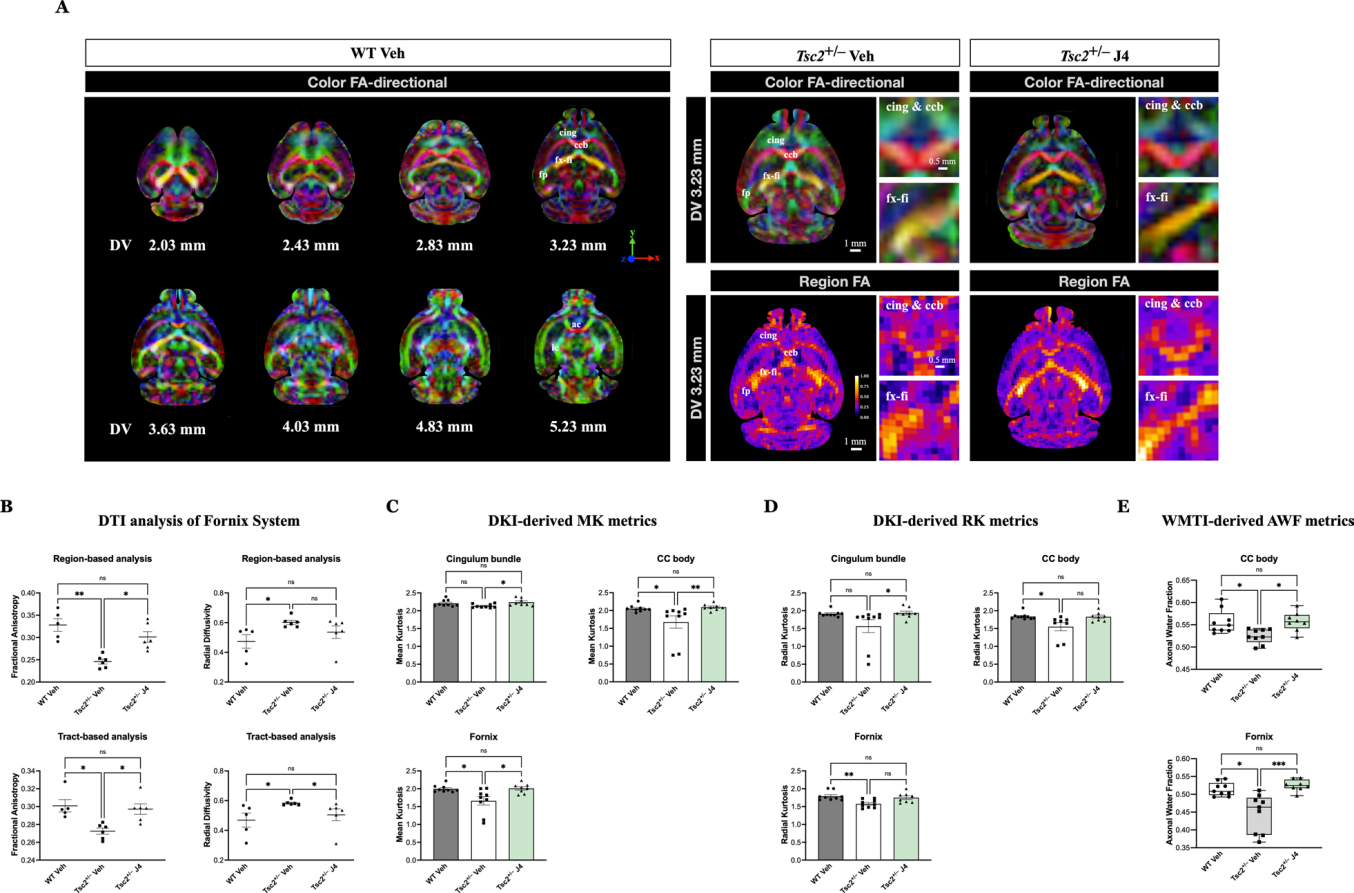
*J4 ameliorated diffusion MRI abnormalities in Tsc2*^+*/–*^* mice.* **A** *Left panel:* Color FA-directional maps from wild-type mice treated with vehicle (WT Veh) at different dorsal–ventral (DV) positions. Fiber tracts directions are indicated by the colors (green: anterior-to-posterior; red: left-to-right; blue: dorsal-to-ventral). *Right panel:* Color FA-directional and region FA maps with a color scale indicating the FA magnitudes in *Tsc2*^+*/–*^ mice treated with vehicle (Veh) or J4: *Tsc2*^+*/–*^ Veh, and *Tsc2*^+*/–*^ J4 groups. Representative horizontal sections are shown at DV 3.23 mm. Major white-matter structures are indicated. **B** DTI analysis of fornix and metrics are compared for WT Veh, *Tsc2*^+*/–*^ Veh, and *Tsc2*^+*/–*^ J4. *Top panel*: Region-based analysis of FA and RD. *Bottom panel*: Tract-based analysis of FA and RD. **C** DKI analysis of the WM regions and MK metrics are shown for each group as indicated. **D** DKI analysis of the WM regions and RK metrics are shown for each group as indicated. **E** WMTI analysis of ccb and fx and AWF metrics are shown for each group as indicated. Statistical analyses were performed using Kruskal–Wallis test followed by Dunn’s post hoc test for multiple comparisons. Data are shown as mean ± SEM.*p < 0.05, **p < 0.01, ***p < 0.001, ****p < 0.0001, *n.s.* not significant, *cing* cingulum bundle, *ccb* body of corpus callosum, *fi* fimbria, *fx* fornix, *fp* posterior forceps, *ac* anterior commissure, *ic* internal capsule, *DTI* diffusion tensor imaging, *DKI* diffusion kurtosis imaging, *WMTI* white matter tract integrity, *FA* fractional anisotropy, *MK* mean kurtosis, *RK* radial kurtosis, *AWF* axonal water fraction

To characterize the WM microstructural abnormalities, we further performed the advanced diffusion MRI, and incorporated DTI, DKI, and WMTI analyses. Since this study focuses on demyelination and myelin integrity, diffusion measures that are greatly affected by the changes in the radial direction of the axonal fiber bundles are shown. The metrics include FA and RD from DTI, mean and MK and RK from DKI, and finally, AWF from WMTI (Fig. 3). See Table 1 for the summary of the diffusion metrics and their statistical significances after multiple comparisons.

**Table 1:** Diffusion MRI metrics comparisons among groups and statistical significances

ROI	dMRI metrics	*P* value^1^	WT Veh v.s. *Tsc2*^+*/–*^ Veh		*Tsc2*^+*/*^ Veh v.s. *Tsc2*^+*/–*^ J4	
			*Tsc2*^+*/–*^ Veh	*P* value^2^	*Tsc2*^+*/–*^ J4	*P* value^3^
Fornix system	Region FA	0.0001***	↓	0.0019**	↑	0.0279*
	Region RD	0.0224*	↑	0.0166*	–	0.4165
	Tracts FA	0.0013**	↓	0.0156*	↑	0.0085**
	Tracts RD	0.0039**	↑	0.0120*	↓	0.0279*
	MK	0.0091**	↓	0.0251*	↑	0.0114*
	RK	0.0067**	↓	0.0051**	↑	0.0439*
	AWF	0.0008***	↓	0.0134*	↑	0.0006***
CC body	MK	0.0022**	↓	0.0251*	↑	0.0016**
	RK	0.0123*	↓	0.0105*	↑	0.0406*
	AWF	0.0084**	↓	0.0325*	↑	0.0083**
Cingulum bundle	MK	0.0287*	–	0.1203	↑	0.0207*
	RK	0.0120*	↓	0.0384*	↑	0.0123*
	AWF	0.7543	–	0.9851	–	0.9431

^1^Kruskal-Wallis test, the non-parametric ANOVA test, was used to determine the statistical significance among the three groups. ^2^Dunn’s test was used as the post hoc multiple comparisons to determine the statistical significance between WT Veh and *Tsc2*^+*/–*^ Veh groups. ^3^Dunn’s test was used as the post hoc multiple comparisons to determine the statistical significance between *Tsc2*^+*/–*^ Veh and *Tsc2*^+*/–*^ J4 groups. * indicates* p* < 0.05, ** indicates *p* < 0.01, *** indicates *p* < 0.001, ns indicates not significant

DTI analysis revealed decreased FA and increased RD in the fornix system of *Tsc2*^+*/–*^ Veh mice, and significantly reversed after J4 treatment (Fig. 3A, B). Correspondingly, we found that *Tsc2*^+*/–*^ Veh mice exhibited decreased MK in WM structures, including body of corpus callosum (ccb) and fornix (fx), but not cingulum bundle (cing). J4 treatment increased MK in these regions (Fig. 3C). In addition, *Tsc2*^+*/–*^ Veh mice showed decreased RK in ccb and fx, but not cing (Fig. 3D), and J4 treatment reversed the MK and RK (Fig. 3C, D). Furthermore, decreased AWF was identified in ccb and fx regions of *Tsc2*^+*/–*^ Veh mice and J4 treatment significantly increased the metric (Fig. 3E).

### J4 enhanced myelin sheath integrity in the WM regions

**Fig. 4:**
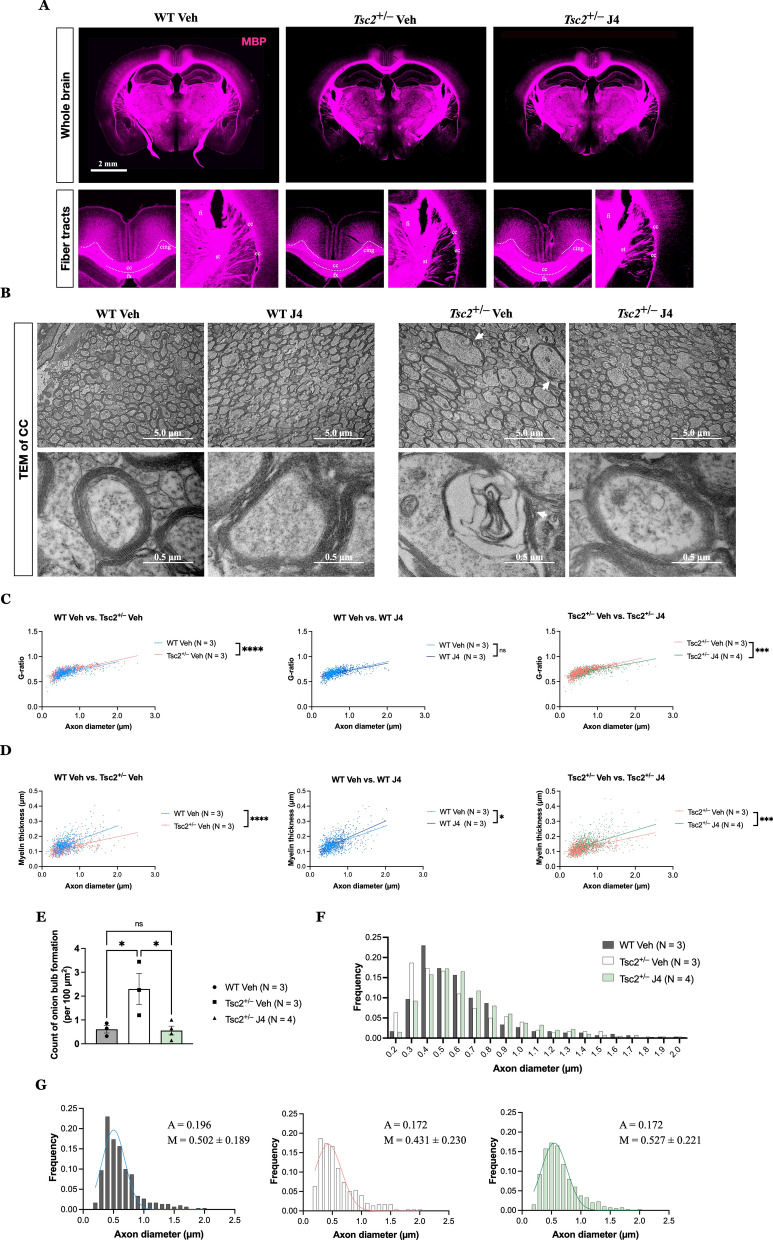
*J4 enhanced myelin sheath integrity in the white matter (WM) regions.* **A** Representative MBP-immunostained coronal brain sections from wild-type (WT) and *Tsc2*^+*/–*^ mice treated with vehicle (Veh) or J4: WT Veh, *Tsc2*^+*/–*^ Veh, and *Tsc2*^+*/–*^ J4 groups. Whole-brain images (top row) show overall myelination patterns, while higher-magnification images (bottom row) highlight major WM tracts. Scale bar (whole brain): 2 mm. **B** TEM images of the corpus callosum from WT Veh, WT J4, *Tsc2*^+*/–*^ Veh, and *Tsc2*^+*/–*^ J4 groups. *Upper panel:* low-magnification views of axonal bundles. *Lower panel:* high-magnification views of individual myelinated axons. Arrows indicate abnormal myelin structures observed in *Tsc2*^+*/–*^ mice. **C** Scatter plot of g-ratio versus axon diameter for all measured axons in the electron micrographs of indicated groups. Each point represents a single axon, and colored regression lines depict fitting lines. **D** Scatter plot of myelin thickness plotted against axon diameter of indicated groups. Each point represents a single axon with regression lines shown for each group. **E** Quantification of the number of myelin bulbs observed in the fornix across groups. Bars represent group means with individual data points from different animals. **F** Histogram showing the frequency distribution of axon diameters measured in the fornix for each group. **G** Axon-diameter distribution plots with fitted Gaussian curves for each group. A and M indicate the fitted amplitude and mean ± SD parameters, respectively. Statistical analyses were performed using linear regression and one-way ANOVA followed by Bonferroni post hoc correction for multiple comparisons. Data are shown as mean ± SEM.*p < 0.05, **p < 0.01, ***p < 0.001, ****p < 0.0001, *n.s.* not significant, *cc* corpus callosum, *ec* external capsule, *fi* fimbra, *fx* fornix, *st* stria terminalis

To further validate the structural alterations observed in the MRI data, we performed fluorescent immunostaining for myelin-basic protein (MBP) to examine myelination in both WM and gray matter (GM) regions. As shown in Fig. 4A, the WM regions did not display any apparent differences in MBP staining when comparing the WT Veh, *Tsc2*^+*/–*^ Veh, and *Tsc2*^+*/–*^ J4 groups. However, transmission electron microscopy (TEM) revealed ultrastructural abnormalities in the myelin sheath of *Tsc2*^+*/–*^ Veh mice (Fig. 4B**)**. Pairwise linear regression comparisons revealed that WT Veh and *Tsc2*^+*/–*^ Veh exhibited similar slopes but significantly different intercepts (F(1, 1215) = 58.06, *p* < 0.0001), indicating a significant upward shift of the g-ratio in *Tsc2*^+*/–*^ Veh. In contrast, *Tsc2*^+*/–*^ Veh and *Tsc2*^+*/–*^ J4 showed significantly different slopes (F(1, 1653) = 15.95, *p* < 0.0001), demonstrating that J4 treatment altered the g-ratio-axon diameter relationship. There were no significant changes in the slopes between WT Veh vs. WT J4 groups (F(1, 974) = 1.986, *p* = 0.1591) (Fig. 4C). Consistently, regression analysis of myelin thickness as a function of axon diameter revealed a flatter slope in *Tsc2*^+*/–*^ Veh mice (F(1, 1214) = 29.59, *p* < 0.0001), confirming reduced myelin thickness across axon calibers. On the other hand, J4 treatment significantly increased the slope (F(1, 1660) = 13.97, *p* = 0.0002), restoring the myelin thickness-axon diameter relationship toward WT Veh control levels, while WT J4 group showed a slight increase of myelin thickness when compared to WT Veh controls (F(1, 974) = 1.986, *p* = 0.0158) (Fig. 4D). Moreover, the occurrence of abnormal myelin structures, such as onion bulb formations, was elevated in *Tsc2*^+*/–*^ Veh mice but markedly reduced following J4 treatment (Fig. 4E; F(2, 7) = 6.727, p = 0.0234, one-way ANOVA; Bonferroni post hoc comparisons: WT Veh vs. *Tsc2*^+*/–*^ Veh, *p* = 0.0375; *Tsc2*^+*/–*^ J4 vs. *Tsc2*^+*/–*^ Veh, *p* = 0.0243). Analysis of axon diameter distribution further revealed that *Tsc2*^+*/–*^ Veh mice exhibited a shift toward smaller axon calibers compared to WT Veh controls (Fig. 4F, G).

### J4 increased myelinated axons in GM regions

**Fig. 5:**
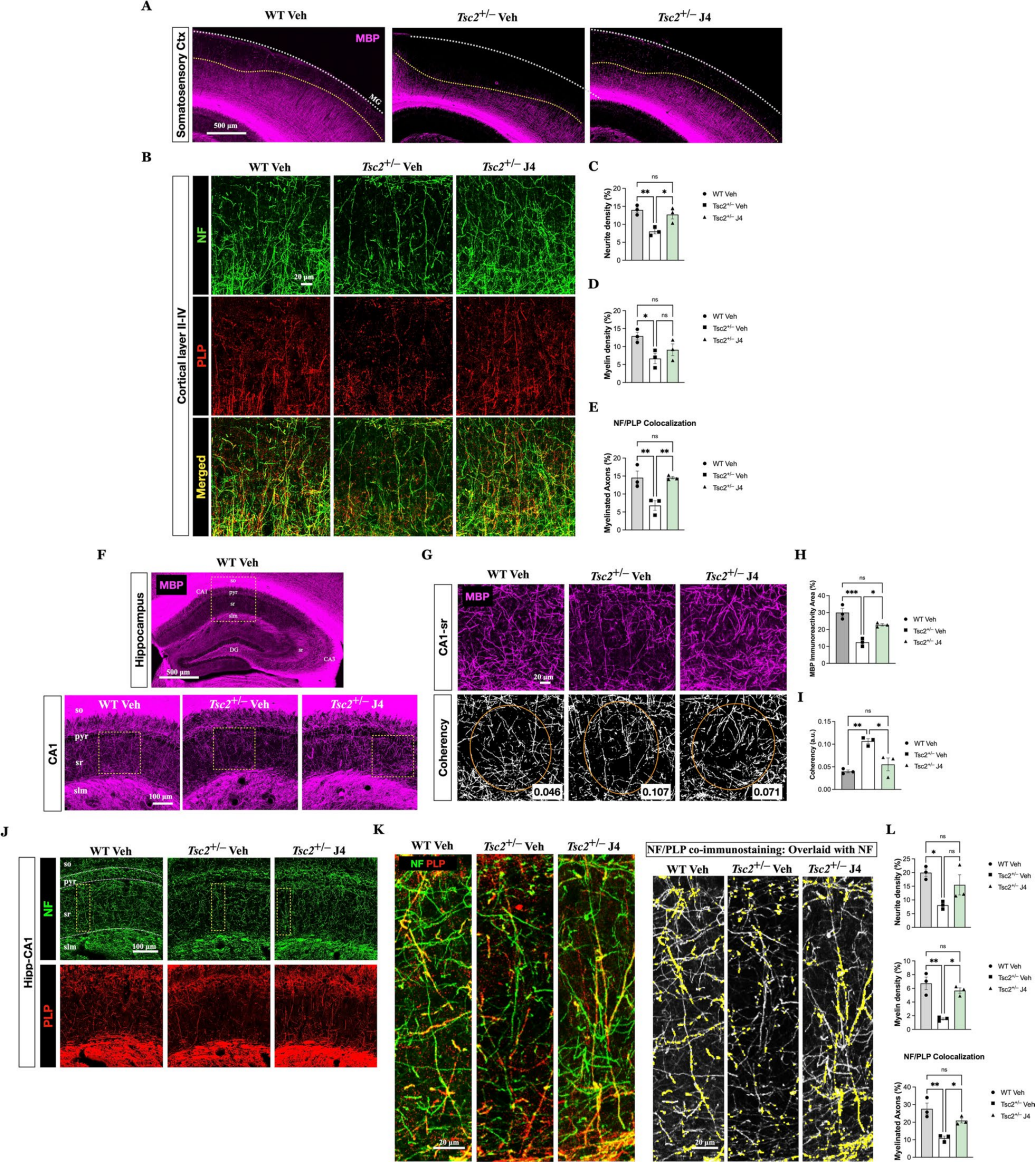
*J4 increased myelinated axons in gray matter (GM) regions.* **A** MBP-immunostained coronal sections of the SS Ctx from wild-type (WT) and *Tsc2*^+*/–*^ mice treated with vehicle (Veh) or J4: WT Veh, *Tsc2*^+*/–*^ Veh, and *Tsc2*^+*/–*^ J4 groups. The dashed lines indicate the extent of myelinated axon projections within the cortex (orange), and the margin of the cortex (white), respectively. Scale bar: 500 μm. **B** Immunofluorescence images of cortical layers II-IV showing staining for SMI-31 (NF) (green) and PLP (red) from each group as indicated. Scale bar: 20 μm. **C** Quantification of SMI-31 (NF)-positive neurite density, **D** PLP-positive myelin density, and **E** SMI-31 (NF)/PLP colocalization in cortical layers II-IV. **F** *Upper panel:* MBP-immunostained coronal sections of the hippocampus from a WT Veh control, showing labeling across subregions CA1-so, CA1-pyr, CA1-sr, CA1-slm, CA3-sr, and DG. Scale bar: 500 μm. *Lower panel:* Higher magnification images of the indicated region by the square from the upper panel. Scale bar: 100 μm. **G** MBP-immunostained images of the CA1-sr (top row). Corresponding coherency maps (bottom row) generated using structure tensor-based analysis (OrientationJ) are shown. **H** Quantification of the MBP-positive immunoreactive area in the CA1-sr. **I** Quantification of coherency values calculated from the OrientationJ.** J** Immunofluorescence images of the hippocampal CA1 region with rectangles showing regions analyzed for co-localization of SMI-31 (NF) (green), and PLP (red). Hippocampal layers including CA1-so, CA1-pyr, CA1-sr, and CA1-slm, are shown. Scale bar: 100 μm. **K** *Left panel:* Higher magnification images of the indicated region by the rectangles in the hippocampal CA1-sr illustrating axonal and myelin co-localization. *Right panel:* representative images processed using Fiji/ImageJ, illustrating the detected co-localized signals (highlighted in yellow) used for quantification. Scale bar: 20 μm. **L** Quantification of SMI-31 (NF)-positive neurite density (*upper panel*), PLP-positive myelin density (*middle panel*), and SMI-31(NF)/PLP colocalized area in the CA1-sr (*bottom panel*). Statistical analyses were performed using one-way ANOVA followed by Bonferroni post hoc correction for multiple comparisons. Data are shown as mean ± SEM.*p < 0.05, ** p < 0.01, ***p < 0.001, **** *p* < 0.0001, *n.s.* not significant, *SS Ctx* somatosensory cortex, *CA1* cornu ammonis 1, *pyr* pyramidal layer, *so* stratum oriens, *sr* stratum radiatum, *slm* stratum, lacunosum-moleculare, *CA3* cornu ammonis 3, *DG* dentate gyrus

In contrast to the WM regions, MBP staining in the GM regions, including the cortex and hippocampus, revealed prominent myelination aberrations in *Tsc2*^+*/–*^ Veh mice compared with WT Veh controls (Fig. 5). Cortical myelination, indicated by MBP immunoreactivity, was pronouncedly reduced and restricted to layer V in *Tsc2*^+*/–*^ Veh mice (Fig. 5A). To further assess axonal myelination, we performed co-immunostaining for SMI-31 (NF) (a marker of phosphorylated neurofilament H, preferably axons) and PLP (a major myelin proteolipid protein) and focused on cortical layers II-IV (Fig. 5B). Confocal images were analyzed quantitatively to measure immunoreactive areas of SMI-31 (NF) and PLP, reflecting neurite density (Fig. 5C) and myelin density (Fig. 5D), respectively. And co-localization of SMI-31 (NF) and PLP immunofluorescent signals was also quantified to show the myelinated axons (Fig. 5E). Quantitative analysis revealed reduced neurite density in *Tsc2*^+*/–*^ Veh mice compared with WT Veh controls (F(2, 6) = 11.40, *p* = 0.0090, one-way ANOVA; Bonferroni post hoc comparisons: WT Veh vs. *Tsc2*^+*/–*^ Veh, *p* = 0.0039). J4 treatment was associated with an increase in neurite density relative to *Tsc2*^+*/–*^ Veh (*Tsc2*^+*/–*^ J4 vs. *Tsc2*^+*/–*^ Veh, *p* = 0.0120), and no significant difference was observed between WT Veh and *Tsc2*^+*/–*^ J4 (*p* = 0.3648).

In contrast, myelin density showed a trend toward reduction in *Tsc2*^+*/–*^ Veh (F(2, 6) = 5.107, *p* = 0.0507), with a significant difference between WT Veh and *Tsc2*^+*/–*^ Veh (*p* = 0.0193). However, while J4 treatment did not significantly alter myelin density compared with *Tsc2*^+*/–*^ Veh (*p* = 0.2642), there was no significant difference between WT Veh and *Tsc2*^+*/–*^ J4 (*p* = 0.1007). Despite these findings, co-localization analysis of NF and PLP suggests that J4 treatment may enhance axon-myelin association (Fig. 5C–E).

Similarly, MBP immunostaining in the hippocampus showed a decrease in immunoreactivity in *Tsc2*^+*/–*^ Veh mice compared with WT Veh controls (Fig. 5F). Quantitative analysis of immunoreactive area within CA1-sr confirmed a significant reduction in MBP expression in *Tsc2*^+*/–*^ Veh mice (Fig. 5G, *upper panel*; Fig. 5H; F(2, 6) = 27.50, *p* = 0.0010, one-way ANOVA; Bonferroni post hoc comparisons: WT Veh vs. *Tsc2*^+*/–*^ Veh, *p* = 0.0010; *Tsc2*^+*/–*^ J4 vs. *Tsc2*^+*/–*^ Veh, *p* = 0.0146; WT Veh vs. *Tsc2*^+*/–*^ J4, *p* = 0.0691). To further assess the organization of myelinated axons, coherency analysis by OrientationJ plugins from Fiji/ImageJ was used to evaluate axonal alignment and complexity. The results showed that J4 treatment increased MBP expression and restored the structural complexity of myelinated axons in the hippocampus (Fig. 5G, *lower panel*; Fig. 5I; F(2, 6) = 15.14, *p* = 0.0045, one-way ANOVA; Bonferroni post hoc comparisons: WT Veh vs. *Tsc2*^+*/–*^ Veh, *p* = 0.0057; *Tsc2*^+*/–*^ J4 vs. *Tsc2*^+*/–*^ Veh, *p* = 0.0203; WT Veh vs. *Tsc2*^+*/–*^ J4, *p* = 0.8172).

Consistently, co-immunostaining for SMI-31 (NF) and PLP in the CA1-sr demonstrated reduced neurite and myelin density in *Tsc2*^+*/–*^ Veh mice (Fig. 5J, K). Quantitative analysis showed a reduction in neurite density (F(2, 6) = 6.701, p = 0.0296; one-way ANOVA; Bonferroni post hoc comparisons: WT Veh vs. *Tsc2*^+*/–*^ Veh, *p* = 0.0332); however, J4 treatment did not significantly alter neurite density compared with *Tsc2*^+*/–*^ Veh (*p* = 0.1923), and no difference was observed between WT Veh and *Tsc2*^+*/–*^ J4 (*p* = 0.6698).

In contrast, myelin density was significantly reduced (F(2, 6) = 15.75, p = 0.0041; WT Veh vs. *Tsc2*^+*/–*^ Veh, *p* = 0.0047), and J4 treatment significantly increased myelin density compared with *Tsc2*^+*/–*^ Veh (*p* = 0.0254), and remained not significantly different from WT Veh (*p* = 0.4752). Furthermore, the proportion of co-localized NF and PLP signals, indicative of myelinated axons, was reduced in *Tsc2*^+*/–*^ Veh mice and increased following J4 treatment (Fig. 5L).

### J4 modulated OL-lineage populations

**Fig. 6:**
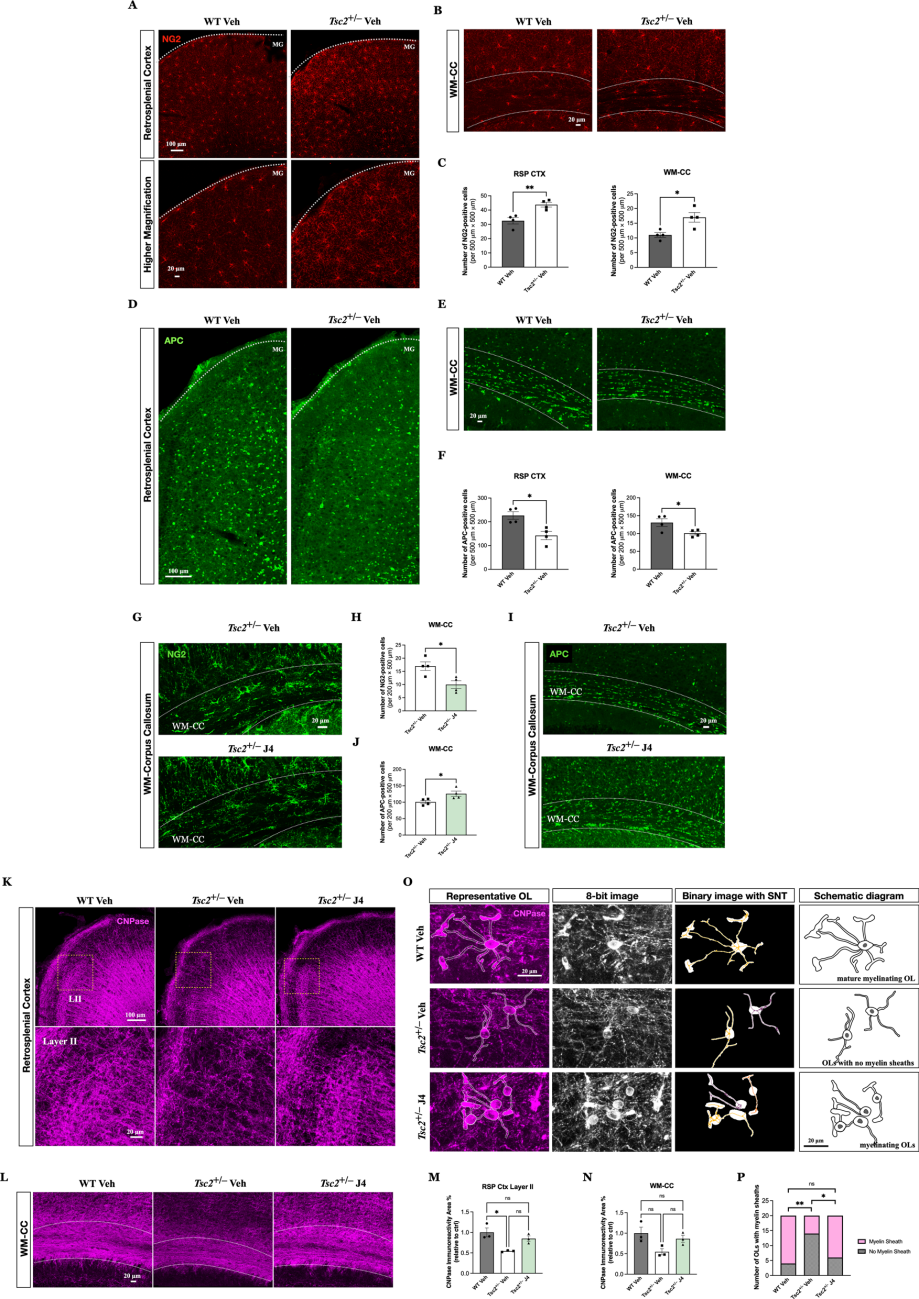
*J4 modulated OL-lineage populations in Tsc2*^+*/–*^* mice.* **A** *Upper panel:* Immunofluorescence images of NG2-positive OPCs in the RSP Ctx from wild-type (WT) and *Tsc2*^+*/–*^ mice treated with vehicle (Veh): WT Veh and *Tsc2*^+*/–*^ Veh groups. The dashed line marks the cortical margin. Scale bar 100 μm. *Lower panel:* Higher magnification images of RSP Ctx. Scale bar: 20 μm. **B** Immunofluorescence images of NG2-positive OPCs in the WM-CC (**C**) Quantification of NG2-positive cell density in the RSP Ctx (*left panel*) and WM-CC (*right panel*). **D** Immunofluorescence images of APC-positive OLs in the RSP Ctx. Scale bar 100 μm. **E** Immunofluorescence images of APC-positive cells in WM-CC. Scale bar: 20 μm. **F** Quantification of APC-positive cells in the RSP Ctx (*left panel*) and WM-CC (*right panel*). **G** Immunofluorescence images of NG2-positive cells in the WM-CC from *Tsc2*^+*/–*^ mice treated with vehicle (Veh) or J4: *Tsc2*^+*/–*^ Veh and *Tsc2*^+*/–*^ J4 groups. The dashed line outlines the white matter structure. Scale bar: 20 μm. **H** Quantification of NG2-positive cells (*upper panel*) and NG2-immunoreactivity (*lower panel*) in the WM-CC.** I** Immunofluorescence images of APC-positive cells in WM-CC. Scale bar: 20 μm. **J** Quantification of APC-positive cells in WM-CC.** K** Immunofluorescence images of CNPase in the RSP Ctx from WT Veh, *Tsc2*^+*/–*^ Veh and *Tsc2*^+*/–*^ J4 groups. **L** Immunofluorescence images of CNPase in the WM-CC. Scale bar: 20 μm. **M** Quantification of CNPase immunoreactivity in RSP cortical layer II. **N** Quantification of CNPase immunoreactivity of WM-CC. **O** Representative images of OLs labeled with CNPase (left), converted to 8-bit images, then to binary image analyzed with SNT and the corresponding reconstructed schematic diagram (right) showing OL process morphology from each group. **P** Quantitative graph showing the ratio of number of OLs with or without myelin sheaths. Statistical analyses were performed using unpaired *t*-test and one-way ANOVA followed by Bonferroni post hoc correction for multiple comparisons. Data are shown as mean ± SEM.*p < 0.05, ** p < 0.01, ***p < 0.001, **** *p* < 0.0001, *n.s.* not significant, *OL* oligodendrocyte, *OPCs* oligodendrocyte precursor cells, *RSP Ctx* retrosplenial cortex, *WM-CC* white matter corpus callosum

To determine whether the oligodendroglial maturation is affected by J4 treatment, we performed immunofluorescence staining using stage-specific OL markers, NG2 for OPCs, APC for immature and mature OLs, and CNPase for mature myelinating processes of OLs. In the RSP Ctx and WM corpus callosum, *Tsc2*^+*/–*^ Veh mice showed an increased number of NG2-positive cells compared with WT Veh controls (*p* = 0.0069 and *p* = 0.0167, unpaired *t*-test), suggesting an accumulation of OPCs (Fig. 6A–C). In parallel, the number of APC-positive cells was reduced in *Tsc2*^+*/–*^ Veh mice compared with WT Veh controls (*p* = 0.0115 and *p* = 0.0469, unpaired *t*–test) in both RSP cortex and WM corpus callosum (Fig. 6D–F).

J4 treatment did not significantly alter OPC number in the RSP Ctx of *Tsc2*^+*/–*^ Veh mice (Supplementary Fig. 2A, B). However, in the WM corpus callosum, J4 treatment reduced the number of NG2-positive cells (*p* = 0.0190, unpaired *t*-test; Fig. 6G, H). Concurrently, the number of APC-positive cells increased in J4-treated *Tsc2*^+*/–*^ mice within the WM corpus callosum (*p* = 0.0376, unpaired *t*-test; Fig. 6I, J). The cortical regions did not show significant changes in OL-lineage after J4 treatment. In contrast, the cingulum bundle, a subcortical WM tract located immediately beneath the cortex, showed a significant increase in APC-positive cells (*p* = 0.010, unpaired *t*–test; Supplementary Fig. 2C–F). Together, these findings suggest that J4 differentially affects OL-lineage populations, with more pronounced changes observed in WM regions than in cortical GM.

Subsequently, we performed immunofluorescence staining for CNPase to assess mature myelinating processes of OLs in layer II-III of the RSP Ctx (Fig. 6K) and WM corpus callosum (Fig. 6L). CNPase immunoreactivity was significantly reduced in the RSP Ctx of *Tsc2*^+*/–*^ Veh mice (F(2, 6) = 9.935, *p* = 0.0125; one-way ANOVA; Bonferroni post hoc comparisons: WT Veh vs. *Tsc2*^+*/–*^ Veh, *p* = 0.0141; Fig. 6M). In contrast, no significant changes were detected in the WM corpus callosum (F(2, 6) = 4.697, *p* = 0.0592; one-way ANOVA; Fig. 6N). J4 treatment showed a trend toward increased CNPase immunoreactivity compared with *Tsc2*^+*/–*^ Veh, although this difference did not reach statistical significance *(p* = 0.0792), and no significant changes were observed between WT Veh and *Tsc2*^+*/–*^ J4 (*p* = 0.5926).

Morphological analysis further indicated that OLs in *Tsc2*^+*/–*^ Veh mice exhibited reduced process extension and fewer myelinating structures. In J4-treated *Tsc2*^+*/–*^ mice, OLs displayed more elaborated processes and increased axonal ensheathment (Fig. 6O). The quantitative analysis of the OL morphology confirmed these differences, showing a significant reduction in the number of OLs with complex myelin structures in *Tsc2*^+*/–*^ Veh compared with WT Veh controls (*p* = 0.0036; Fisher’s exact test), and an increase in J4-treated *Tsc2*^+*/–*^ mice relative to *Tsc2*^+*/–*^ Veh (*p* = 0.0256), with no significant difference between WT Veh and *Tsc2*^+*/–*^ J4 (*p* = 0.7164; Fig. 6P).

### *J4 mitigated the excessive expression of pS6 and cFos in Tsc2*^+*/–*^* mice*

**Fig. 7:**
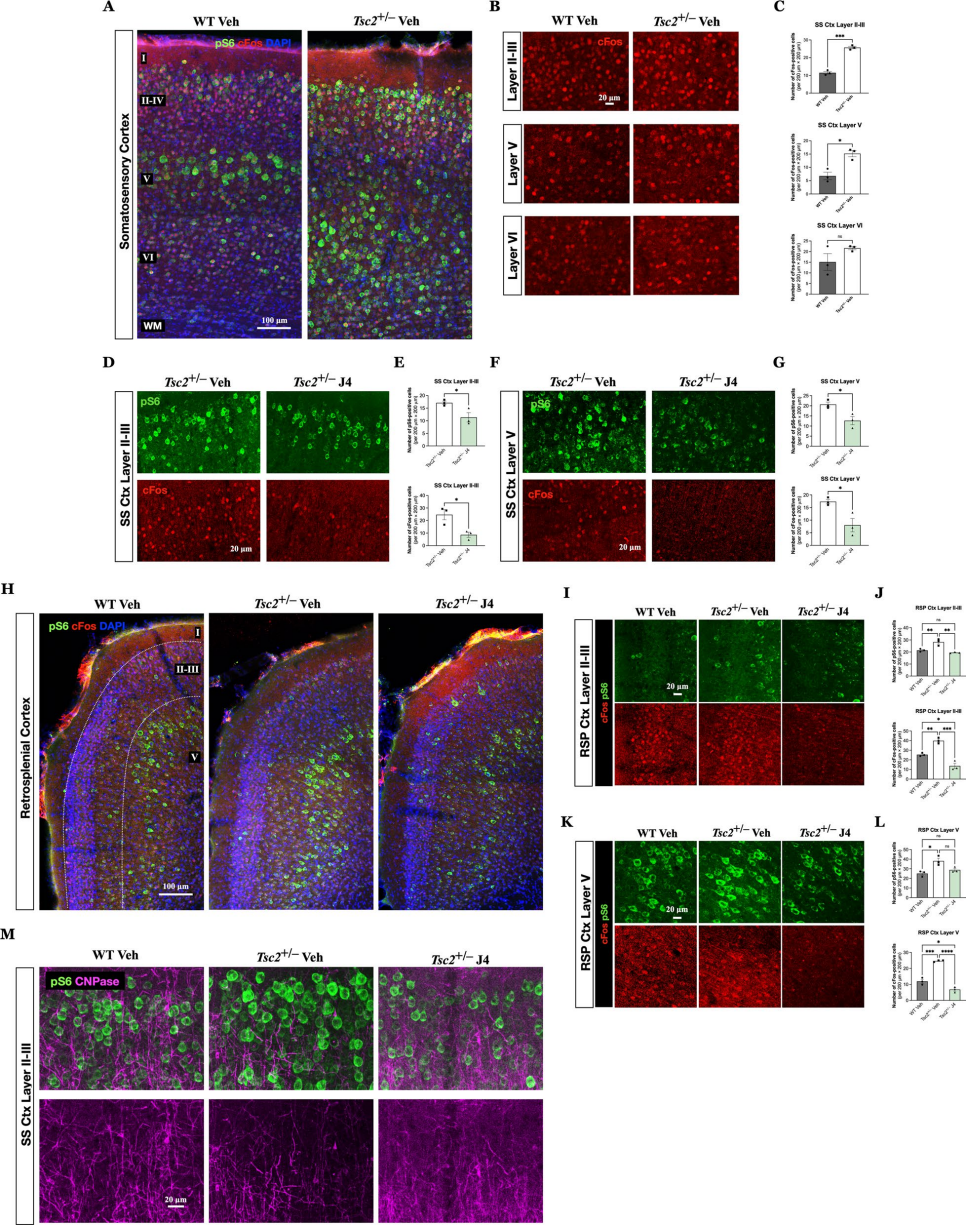
*J4 mitigated the excessive expression of pS6 and cFos in Tsc2*^+*/–*^* mice.* **A** Immunofluorescence images of the SS Ctx in cortical layers (I, II-III, V, and VI) and the WM showing pS6 (green), cFos (red), and DAPI (blue) labeling in wild-type (WT) and *Tsc2*^+*/–*^ mice treated with vehicle (Veh): WT Veh and *Tsc2*^+*/–*^ Veh groups.** B** Immunofluorescence images of cFos in cortical layers II-III, V, and VI for better comparison between WT and *Tsc2*^+*/–*^ mice. **(C)** Quantification of cFos immunoreactivity in cortical layers II-III (top), V (middle), and VI (bottom). **D** Immunofluorescence images of pS6 (green) and cFos (red) in the SS cortical layer II-III from *Tsc2*^+*/–*^ mice treated with vehicle (Veh) or J4: *Tsc2*^+*/–*^ Veh and *Tsc2*^+*/–*^ J4 groups. **E** Quantification of pS6-positive cells (top) and cFos-positive cells (bottom) in SS cortical layer II-III. **F** Immunofluorescence images of pS6 (green) and cFos (red) in the SS cortical layers V. **G** Quantification of pS6-positive cells (top) and cFos-positive cells (bottom) in SS layer V. **H** Immunofluorescence images of RSP cortical layers (I, II-III, and V) showing pS6 (green), cFos (red) and DAPI (blue) labeling in WT Veh, *Tsc2*^+*/–*^ Veh and *Tsc2*^+*/–*^ J4 groups.** I** Immunofluorescence images of pS6 (green) and cFos (red) in the RSP cortical layer II-III. **J** Quantification of pS6-positive cells (top) and cFos-positive cells (bottom) in RSP layer II-III. **K** Immunofluorescence images of pS6 (green) and cFos (red) in the RSP cortical layer V. **L** Quantification of pS6-positive cells (top) and cFos-positive cells (bottom) in RSP layer V. Statistical analyses were performed using unpaired *t*-test and one-way ANOVA followed by Bonferroni post hoc correction for multiple comparisons. Data are shown as mean ± SEM.*p < 0.05, ** p < 0.01, ***p < 0.001, **** *p* < 0.0001, *n.s.* not significant, *SS Ctx* somatosensory cortex, *RSP Ctx* retrosplenial cortex, *WM* white matter

In *Tsc2*^+*/–*^ Veh mice, we observed markedly increased expression of both pS6 and cFos in the SS Ctx compared with WT controls (Fig. 7A). In WT brains, pS6 expression was most prominent in cortical layer V, as shown by the co-localization with the layer V marker, Ctip2 (Supplementary Fig. 3). This result shows its normal laminar distribution which is consistent with previous reports [25, 57, 58]. However, in *Tsc2*^+*/–*^ Veh mice, while cortical lamination remains intact, pS6 immunoreactivity shows a more dispersed distribution across cortical layers, as revealed by co-immunostaing with Ctip2 (Supplementary Fig. 3). Quantitative analysis of cFos-positive cells across cortical layers II-III, V, and VI revealed a significant increase in the number of cFos-positive cells in layers II-III and V (*p* = 0.0002 and *p* = 0.0104, unpaired *t*-test), with no changes in layer VI (*p* = 0.1788, respectively), suggesting widespread neuronal hyperactivity associated with mTOR pathway dysregulation (Fig. 7B, C).

To determine whether J4 treatment modulates neuronal hyperactivity, we examined pS6 and cFos expression in the SS and RSP cortices (Fig. 7D–L). In the SS Ctx, J4 treatment was associated with a reduction in neuronal activity in *Tsc2*^+*/–*^ Veh mice, as indicated by decreased pS6- and cFos-positive cells in layer II–III (*p* = 0.0430 and *p* = 0.0238, respectively) and layer V (*p* = 0.0274 and *p* = 0.0287, respectively), compared with *Tsc2*^+*/–*^ Veh mice (Fig. 7D–G).

In the RSP Ctx, quantitative analysis revealed significant increases in both pS6- and cFos-positive cells in *Tsc2*^+*/–*^ Veh mice compared with WT Veh controls in layer II–III (pS6: F(2, 6) = 20.51, *p* = 0.0021; WT Veh vs. *Tsc2*^+*/–*^ Veh, *p* = 0.0094; cFos: F(2, 6) = 43.51, *p* = 0.0003; WT Veh vs. *Tsc2*^+*/–*^ Veh, *p* = 0.0064) and layer V (pS6: F(2, 6) = 9.69, *p* = 0.0132; WT Veh vs. *Tsc2*^+/−^ Veh, *p* = 0.0156; cFos: F(2, 6) = 93.94, *p* < 0.0001; WT Veh vs. *Tsc2*^+/−^ Veh, *p* = 0.0002) (Fig. 7H–L).

J4 treatment significantly reduced pS6- and cFos-positive cells in layer II–III (pS6: *p* = 0.0027; cFos: *p* = 0.0003) and cFos-positive cells in layer V (*p* < 0.0001), while pS6 reduction in layer V showed a trend but did not reach statistical significance (*p* = 0.0688). Notably, no significant difference was observed between WT Veh and *Tsc2*^+*/–*^ J4 groups for pS6 in either layer II–III or layer V (*p* = 0.6875 and *p* = 0.7788, respectively), whereas cFos levels in *Tsc2*^+*/–*^ J4 mice were significantly reduced compared with WT Veh controls (layer II–III, *p* = 0.0176; layer V, *p* = 0.0261).

Furthermore, co-immunostaining for CNPase and pS6 in cortical layer II–III revealed reduced CNPase expression accompanied by elevated pS6 immunoreactivity in *Tsc2*^+*/–*^ Veh mice, indicating hyperactivation of mTOR signaling and reduced oligodendroglial myelinating processes (Fig. 7M). Quantitative analysis confirmed a reduction in mature, myelinating oligodendrocytes in *Tsc2*^+*/–*^Veh mice, whereas J4 treatment was associated with increased oligodendroglial myelin sheath formation and a concurrent reduction in pS6-positive cells (Fig. 6P; Fig. 7E).

## Discussion

### Behavioral improvements following J4 treatment

Our study investigated the therapeutic potential of the novel compound, J4, in *Tsc2*^+*/–*^ mice, a model that recapitulates cognitive deficits and anxiety-like behaviors characteristic of TSC-associated neuropsychiatric symptoms. Consistent with clinical observations that individuals with TSC commonly exhibit intellectual disability and neuropsychiatric impairments, *Tsc2*^+*/–*^ mice demonstrated impaired performance in the NOR task and increased anxiety-like behavior in the OFT. Notably, J4-treated *Tsc2*^+*/–*^ mice showed substantial improvement in object recognition memory and increased center exploration, indicating a functional rescue of neural circuits involved in recognition memory and emotional regulation (Fig. 1). Given that neural circuit function critically depends on axonal fiber tracts connecting brain regions—structures composed predominantly of WM—targeting WM plasticity during postnatal development represents a promising therapeutic strategy. Our previous work demonstrated that subtle alterations in WM microstructure and myelination complexity in the TSC mouse model can be restored by a natural mTOR inhibitor [51, 53]. Together with findings from other groups [59, 60], these results suggest that WM and myelination deficits in TSC are reversible, and that oligodendrogenesis and WM neuroplasticity remain active even in adulthood, providing an opportunity for therapeutic intervention.

### Diffusion MRI reveals microstructural WM deficits in Tsc2^+/–^ mice

Although *Tsc2*^+*/–*^ mice displayed NAWM on T2-weighted MRI and LFB staining, diffusion MRI revealed microstructural abnormalities (Fig. 2, 3). Specifically, we observed reduced FA, MK, RK, and AWF, alongside increased RD in the WM regions, indicative of compromised axonal organization or myelin integrity that is not detected by conventional MRI. This discrepancy parallels clinical findings in TSC patients, in which  WM anomalies are often undetectable on routine MRI scans despite cognitive impairment. Hence, DTI has been widely applied to assess the WM pathologies and their association with neurological comorbidity in individuals with TSC [56, 61, 62]. In consistent with both human and animal studies reporting decreased FA and increased RD in the corpus callosum [22, 51, 53, 63, 64], our present study demonstrated similar diffusion alterations in the fiber bundles. In addition to the corpus callosum, which is a major component of the lateral forebrain bundle system, we showed that medial forebrain bundle system of *Tsc2*^+*/–*^ mice is also abnormal. Specifically, the fornix system, which is a C-shaped nerve fiber bundle that connects the hippocampus to other parts of the brain, exhibited reduced FA and elevated RD in *Tsc2*^+*/–*^ mice, indicating WM microstructural aberrations in brain structures that are critical for learning and memory contribute to the neuropsychiatric disorders in *Tsc2*^+*/–*^ mice (Fig. 3).

To further enhance sensitivity to subtle microstructural changes, we employed DKI, which captures non-Gaussian water diffusion and reflects brain tissue complexity and/or heterogeneity [65]. We observed decreased MK and RK in both the corpus callosum body and fornix of *Tsc2*^+*/–*^ mice. These reductions in kurtosis metrics align with the myelin sheath abnormalities identified by TEM, including increased g-ratio values and impaired myelin compaction (Fig. 4B-G), supporting the utility of DKI as a sensitive measure of underlying myelin pathology. In contrast, MK and RK changes were not detected in the fimbria, likely due to limited spatial resolution or partial volume effects in this small region within the fornix system. However, WMTI analysis successfully detected architectural alterations, as evidenced by reduced AWF in *Tsc2*^+*/–*^ mice (Fig. 3E). Nevertheless, our data indicate an overall pattern of aberrant myelination and axons in the WM of *Tsc2*^+*/–*^ mice. Importantly, J4 treatment normalized robustly these diffusion metrics and myelin sheath defects, supporting that improved WM microstructure underlies, at least in part, the behavioral rescue observed in J4-treated animals. These findings highlight the sensitivity of advanced diffusion MRI metrics in detecting treatment-responsive changes in myelin and axonal integrity.

### The interplay between mTOR signaling and myelination

Although MBP immunostaining appeared grossly normal in WM, TEM captured deficits in myelin compaction that immunohistochemistry could not detect (Fig. 4). The ultrastructural images from TEM indicate that Tsc2 haploinsufficiency disrupts myelin assembly. Furthermore, we also demonstrated that disrupted WM structures and GM myelination in *Tsc2*^+*/–*^ mice were accompanied by impaired maturation of OL-lineage cells (Figs. 5, 6). Our results are consistent with previous studies. One study has documented the splitting myelin sheaths in nanofiber culture using OLs derived from the TSC patient, in addition to impaired development of OPCs into differentiated myelinating OLs [40]. Indeed, studies targeting *Tsc1* or *Tsc2* deletion specifically in OL-lineage cells have demonstrated that the excessive mTOR activity disrupts both the timing and quality of myelination, resulting in thinner, or poorly compacted myelin sheaths and improper myelinogenesis [66, 67]. Similarly, neuronal *Tsc1* or *Tsc2* deletion leads to alterations in oligodendrogial development, hypomyelination, and reduced MBP expression [39, 68, 69], all of which negatively impact myelin formation. The present study further supports the notion that dysregulation of the mTOR pathway directly influences oligodendroglial maturation and myelin sheath formation.

Many mechanisms have been proposed to explain why hyperactivation of mTORC1—the central regulator of the mTOR pathway—leads to hypomyelination and myelin sheath abnormalities, rather than the excessive myelin growth that might intuitively be expected. These mechanisms include the induction of endoplasmic reticulum (ER) stress and activation of the unfolded protein response in OL-lineage cells [70], as well as feedback suppression of the Mek-Erk1/2 and/or PI3K-Akt pathways and additional mTORC1-independent targets disrupted by loss of the TSC complex [71]. Despite these complexities, studies investigating the interplay between mTOR signaling and myelination converge on several fundamental principles: myelin formation is highly metabolically demanding during development, a continuous supply of myelin components is required throughout adulthood to sustain adaptive myelination, and the remyelination can be rapidly initiated following injury [71].

Neuropathological studies in both mouse models of TSC and patient tissue further support a metabolic basis to myelin abnormalities, revealing an accumulation of organelles, including mitochondria, due to defective autophagic flux [72–75]. In fact, neuronal hyperactivation of mTORC1 caused by *Tsc2* deficiency has been shown to impair axonal mitochondrial dynamics [76]. Mitochondrial dysfunction has profound consequences for myelin formation and WM integrity. Disrupted neuronal mitochondrial homeostasis can lead to energy deprivation that alters neuro-glia interaction, thereby impairing processes required for proper myelination. In OLs, mitochondrial impairment interrupts lipid synthesis and metabolic support, both which are essential for generating and maintaining compact myelin sheaths [77].

Given these metabolic deficiencies in TSC, the ability of J4 to mediate cellular energy balance [47] provides a possible mechanistic link to its therapeutic effects in *Tsc2*^+*/–*^ mice. Previously, J4 has been shown to improve mitochondrial function and energy balance by normalizing adenosine levels and suppressing overactivated AMPK—an energy sensor—in a tauopathy model characterized by metabolic stress. By improving mitochondrial efficiency and reducing energy deprivation, J4 may restore the metabolic conditions necessary for proper lipid synthesis, process extension and myelin compaction in OLs. Additionally, improved neuronal mitochondrial function may normalize axon-glia signaling, further promoting myelination. Thus, the capacity of J4 to maintain cellular energy homeostasis offers a plausible mechanism through which it ameliorates the myelin abnormalities observed in *Tsc2*^+*/–*^ mice. In addition, J4 also suppressed the excessive pS6 expression in *Tsc2*^+*/–*^ mice (Fig. 7), hence we cannot exclude the possibility that J4 directly exerts its effects by attenuating mTORC1 overactivation. This may act in parallel with its influence on cellular energy homeostasis. However, more comprehensive investigations are needed to determine the extent to which J4 directly regulates mTOR signaling or acting through other pathways.

### J4 reduces cortical neuronal hyperactivity and modulates OL-lineage populations in WM

**Fig. 8:**
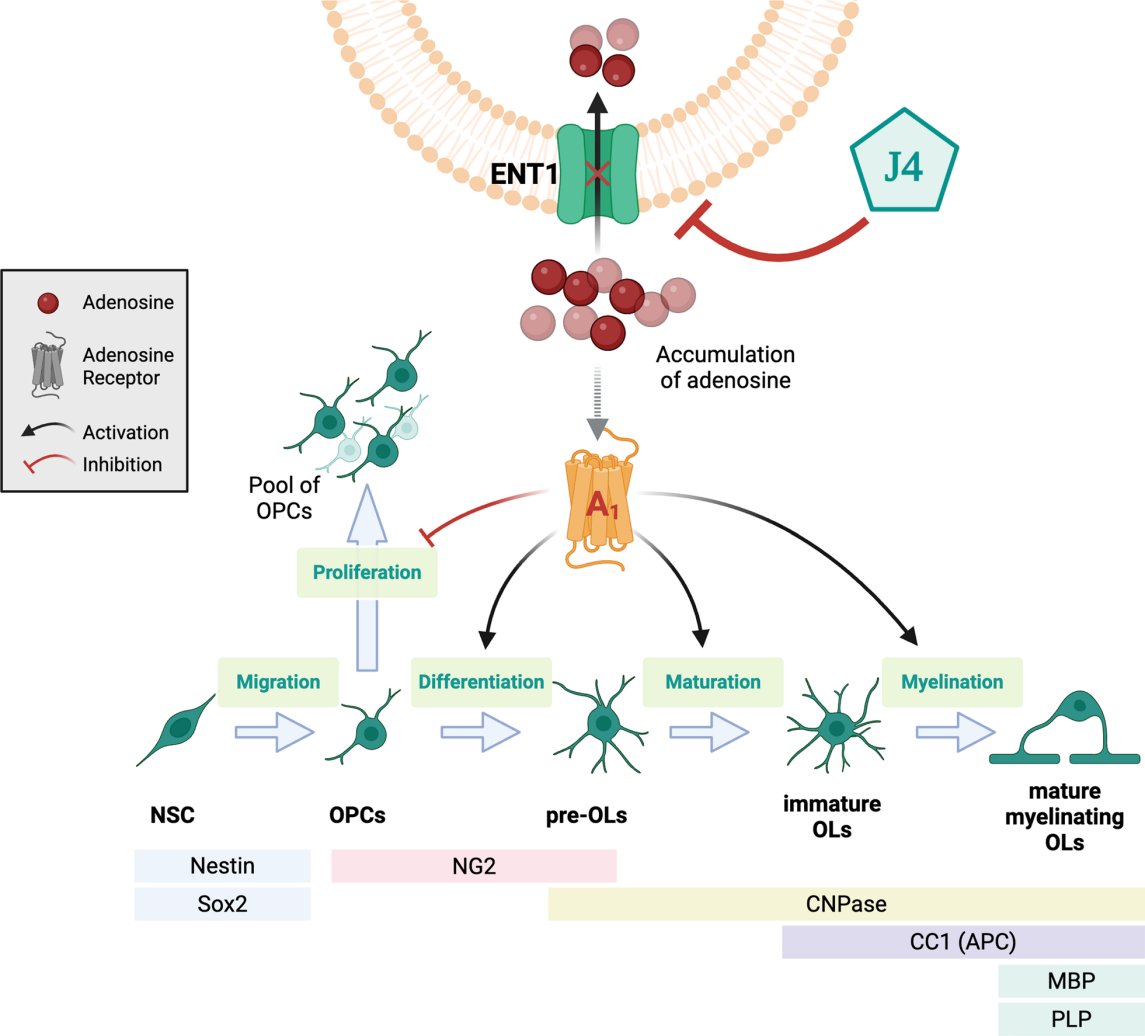
*Proposed mechanism by which ENT1 inhibition by J4 enhances oligodendrocyte maturation.* J4 inhibits ENT1, leading to reduced adenosine uptake and consequent accumulation of extracellular adenosine. Activation of adenosine receptor A_1_ has been demonstrated to differentially regulate distinct stages of the OL lineage. Activation of this receptor promotes NSC migration, OPC differentiation, and OL maturation, simultaneously inhibiting OPC proliferation. Representative markers for each developmental stage are shown Nestin and Sox2 for NSCs; NG2 for OPCs; CNPase for pre-OLs, immature and mature OLs; APC for immature and mature OLs; and MBP and PLP for mature myelinating OLs [79]. In J4-treated *Tsc2*^+*/–*^ mice, extracellular adenosine is presumed to be elevated, leading to preferential activation of A_1_ receptors, thereby promoting OPC differentiation, maturation, and myelination as illustrated by the arrows. NSCs, neural stem cells; OPCs, oligodendrocyte precursor cells; OLs, oligodendrocytes. Created with Biorender.com

Importantly, J4 has been shown to modulate extracellular adenosine levels by inhibiting ENT1 in various experimental models [45–47]. Adenosine, acting through its receptors, plays a well-established role in regulating OPC migration, differentiation, maturation, and myelination [42, 78]. Integrating our findings with the known involvement of adenosine signaling in oligodendroglial development [79], we proposed a working model of J4’s mechanism of actions in the process of OPC differentiation, OL maturation, and myelination, as illustrated in Fig. 8. Since adenosine has a higher binding affinity for A_1_ receptors, which are more abundantly expressed than other adenosine receptors [80–82], extracellular adenosine presumed to be accumulated after J4 treatment may preferentially activate A_1_ receptors, thereby contributing to OPC differentiation into pre-OLs, as depicted in the diagram. The observed improvements in WM structures may be partially explained by enhanced OL lineage progression following J4 treatment. J4 significantly reduced the immunoreactivity and the number of NG2-positive cells in the corpus callosum, and concomitantly increased the number of APC-positive cells in the WM regions, indicating a shift toward OL maturation (Fig. 6G–J, Supplementary Fig. 2E, F). However, no significant differences in CNPase immunoreactivity were detected in the corpus callosum in all 3 groups (Fig. 6L, M). In mature OLs, CNPase is predominantly localized to OL processes and myelin-associated structure rather than clearly defined cell bodies; therefore, area-based CNPase quantification may not sensitively capture changes in mature OL abundance, especially in densely myelinated white matter.

On the other hand, CNPase immunostaining demonstrated reduced cortical myelination (Fig. 6K) and a lower proportion of OLs displaying myelin sheath wrapping in Tsc2-deficient mice (Fig. 6O). This phenotype was restored following J4 treatment (Fig. 6P), while NG2 and APC immunostaining revealed no significant changes in cortical OL-lineage cell populations after J4 treatment. This regional discrepancy suggests that oligodendroglial response to J4 may differ between gray and white matter. One possible explanation is that the effects of J4 on cortical OLs may be mediated through modulation of neuronal activity. This is supported by that the elevated pS6 and cFos immunoreactivity observed in the cortex of *Tsc2*^+*/–*^ mice (Fig. 7A–L), were both reduced following J4 treatment. Consistent with this notion, previous studies have shown that neuronal activity can regulate OL function and myelin remodeling through activity-dependent neuron-glia signaling [83, 84]. Importantly, aberrantly increased neuronal activity induced by seizures has been shown to impair OL maturation and myelination, leading to increased OPC proliferation, reduced mature  OLs, and decreased myelination [85]. Therefore, the restoration of cortical myelin sheath wrapping by J4 may occur due to the normalization of neuronal activity. Whereas in WM regions, J4 appears to affect oligodendrolial lineage dynamics, as evidenced by reduced NG2-positive OPCs and increased APC-positive oligodendroglial cells.

Nevertheless, our findings suggest that Tsc2 haploinsufficiency disrupts oligodendroglial homeostasis and myelin integrity in both WM and GM. J4 treatment may facilitate OL-lineage progression in WM regions while enhancing GM myelin sheath formation and wrapping through neuron-glia interactions. Taken together, the combined effects of J4 on OL-lineage cells and neuronal signaling may contribute to the restoration of myelin microstructure in both WM and GM, thereby improving of cognitive and emotional behavioral outcomes.

### Limitations of this present study

Several limitations should be acknowledged in the present study. First, as only male *Tsc2*^+*/–*^mice were used, the findings of this study are limited to male animals. Second, *Tsc2*^+*/–*^ mice display a relatively mild phenotypes compared with more severe *Tsc1* or *Tsc2* conditional knockout models, which may partly account for the discrepancies observed between our diffusion MRI findings and the more pronounced histopathological alterations reported previously. Third, while our data suggest that J4 may indirectly modulate mTOR signaling and dampen neuronal hyperactivity, the precise mechanistic relationship between these pathways and myelination remains unclear and warrants further investigation. Fourth, our findings are consistent with enhanced oligodendroglial maturation in WM regions following J4 treatment, yet the current data do not directly demonstrate lineage progression from OPCs to mature oligodendrocytes. Finally, although J4 is known to elevate extracellular adenosine levels through ENT1 inhibition, the direct measurement of adenosine and the downstream molecular pathways through which adenosine receptor signaling influences oligodendroglial maturation are not yet fully defined in this present study and require further investigation.

## Conclusion

In this study, we evaluated the potential therapeutic effects of J4 on TAND-like phenotypes in a mouse model of TSC. Our results indicated that J4 treatment improved cognitive dysfunction and anxiety-like behavior in *Tsc2*^+*/–*^ mice. Moreover, by incorporating non-invasive neuroimaging modalities, diffusion kurtosis and diffusion tensor imaging, we found that J4 treatment was able to reverse the overall WM microstructural abnormalities in *Tsc2*^+*/–*^ mice. Evidently, J4 treatment can also change the myelin and axonal architectures by facilitating the enhancement of mature, myelinating OLs. Our study provides a new alternative approach for treating TSC-related cognitive impairment and anxiety-like behavior.

## Supplementary Information

Supplementary Material 1.

Supplementary Material 2.

Supplementary Material 3.

Supplementary Material 4.

## Data Availability

The data that support the findings of this study are available on request to the corresponding author.

## References

[1] Henske EP, et al. Tuberous sclerosis complex. Nat Rev Dis Primers. 2016;2:16035. 27226234 10.1038/nrdp.2016.35

[2] Northrup HKM, Pearson DA et al. Tuberous Sclerosis Complex, in GeneReviews®. 1999

[3] Marom D. Genetics of tuberous sclerosis complex: an update. Childs Nerv Syst. 2020;36(10):2489–96. 32761379 10.1007/s00381-020-04726-z

[4] Chu-Shore CJ, et al. The natural history of epilepsy in tuberous sclerosis complex. Epilepsia. 2010;51(7):1236–41. 20041940 10.1111/j.1528-1167.2009.02474.x PMC3065368

[5] Nabbout R, et al. Epilepsy in tuberous sclerosis complex: findings from the TOSCA Study. Epilepsia Open. 2019;4(1):73–84. 30868117 10.1002/epi4.12286 PMC6398114

[6] de Vries PJ, et al. TSC-associated neuropsychiatric disorders (TAND): findings from the TOSCA natural history study. Orphanet J Rare Dis. 2018;13(1):157. 30201051 10.1186/s13023-018-0901-8 PMC6131901

[7] Prather P, de Vries PJ. Behavioral and cognitive aspects of tuberous sclerosis complex. J Child Neurol. 2004;19(9):666–74. 15563012 10.1177/08830738040190090601

[8] Gillberg IC, Gillberg C, Ahlsén G. Autistic behaviour and attention deficits in tuberous sclerosis: a population-based study. Dev Med Child Neurol. 1994;36(1):50–6. 8132114 10.1111/j.1469-8749.1994.tb11765.x

[9] Spurling Jeste S, et al. Early developmental trajectories associated with ASD in infants with tuberous sclerosis complex. Neurology. 2014;83(2):160–8. 24920850 10.1212/WNL.0000000000000568 PMC4117170

[10] Leclezio L, et al. Pilot validation of the tuberous sclerosis-associated neuropsychiatric disorders (TAND) checklist. Pediatr Neurol. 2015;52(1):16–24. 25499093 10.1016/j.pediatrneurol.2014.10.006

[11] de Vries PJ, et al. International consensus recommendations for the identification and treatment of tuberous sclerosis complex-associated neuropsychiatric disorders (TAND). J Neurodev Disord. 2023;15(1):32. 37710171 10.1186/s11689-023-09500-1 PMC10503032

[12] Ehninger D, et al. Reversal of learning deficits in a Tsc2+/- mouse model of tuberous sclerosis. Nat Med. 2008;14(8):843–8. 18568033 10.1038/nm1788 PMC2664098

[13] Sato A, et al. Rapamycin reverses impaired social interaction in mouse models of tuberous sclerosis complex. Nat Commun. 2012;3:1292. 23250422 10.1038/ncomms2295 PMC3535343

[14] Zeng LH, et al. Rapamycin prevents epilepsy in a mouse model of tuberous sclerosis complex. Ann Neurol. 2008;63(4):444–53. 18389497 10.1002/ana.21331 PMC3937593

[15] Randell E, et al. The use of everolimus in the treatment of neurocognitive problems in tuberous sclerosis (TRON): study protocol for a randomised controlled trial. Trials. 2016;17:398. 27515417 10.1186/s13063-016-1446-6 PMC4981993

[16] Krueger DA, et al. Everolimus for treatment of tuberous sclerosis complex-associated neuropsychiatric disorders. Ann Clin Transl Neurol. 2017;4(12):877–87. 29296616 10.1002/acn3.494 PMC5740257

[17] Overwater IE, et al. A randomized controlled trial with everolimus for IQ and autism in tuberous sclerosis complex. Neurology. 2019;93(2):e200–9. 31217257 10.1212/WNL.0000000000007749

[18] Northrup H, et al. Updated international tuberous sclerosis complex diagnostic criteria and surveillance and management recommendations. Pediatr Neurol. 2021;123:50–66. 34399110 10.1016/j.pediatrneurol.2021.07.011

[19] Ho CN, R.G., Valentine JE, Rosbeck KL, Roberds SL. The voice of the patient: a report from the tuberous sclerosis alliance externtally-led patient-focused drug development meeting*.* 2018.

[20] de Vries PJ. Targeted treatments for cognitive and neurodevelopmental disorders in tuberous sclerosis complex. Neurotherapeutics. 2010;7(3):275–82. 20643380 10.1016/j.nurt.2010.05.001 PMC5084231

[21] Tye C, et al. Long-term cognitive outcomes in tuberous sclerosis complex. Dev Med Child Neurol. 2020;62(3):322–9. 31538337 10.1111/dmcn.14356 PMC7027810

[22] Baumer FM, et al. Corpus Callosum white matter diffusivity reflects cumulative neurological comorbidity in tuberous sclerosis complex. Cereb Cortex. 2017;28(10):3665–72. 10.1093/cercor/bhx247 PMC6132277 29939236

[23] Gąssowska-Dobrowolska M, et al. Microtubule cytoskeletal network alterations in a transgenic model of tuberous sclerosis complex: relevance to autism spectrum disorders. Int J Mol Sci. 2023. 10.3390/ijms24087303. 37108467 10.3390/ijms24087303 PMC10138344

[24] Lewis WW, et al. Impaired language pathways in tuberous sclerosis complex patients with autism spectrum disorders. Cereb Cortex. 2013;23(7):1526–32. 22661408 10.1093/cercor/bhs135 PMC3673171

[25] Meikle L, et al. A mouse model of tuberous sclerosis: neuronal loss of Tsc1 causes dysplastic and ectopic neurons, reduced myelination, seizure activity, and limited survival. J Neurosci. 2007;27(21):5546–58. 17522300 10.1523/JNEUROSCI.5540-06.2007 PMC6672762

[26] Sosunov AA, et al. Tuberous sclerosis: a primary pathology of astrocytes? Epilepsia. 2008;49(Suppl 2):53–62. 18226172 10.1111/j.1528-1167.2008.01493.x

[27] Wong M. The role of glia in epilepsy, intellectual disability, and other neurodevelopmental disorders in tuberous sclerosis complex. J Neurodev Disord. 2019;11(1):30. 31838997 10.1186/s11689-019-9289-6 PMC6913020

[28] Peters JM, et al. Diffusion tensor imaging and related techniques in tuberous sclerosis complex: review and future directions. Future Neurol. 2013;8(5):583–97. 24489482 10.2217/fnl.13.37 PMC3904372

[29] Krishnan ML, et al. Diffusion features of white matter in tuberous sclerosis with tractography. Pediatr Neurol. 2010;42(2):101–6. 20117745 10.1016/j.pediatrneurol.2009.08.001 PMC2831465

[30] Griffiths PD, Bolton P, Verity C. White matter abnormalities in tuberous sclerosis complex. Acta Radiol. 1998;39(5):482–6. 9755694 10.1080/02841859809172211

[31] Carson RP, et al. Hypomyelination following deletion of Tsc2 in oligodendrocyte precursors. Ann Clin Transl Neurol. 2015;2(12):1041–54. 26734657 10.1002/acn3.254 PMC4693589

[32] Bradl M, Lassmann H. Oligodendrocytes: biology and pathology. Acta Neuropathol. 2010;119(1):37–53. 19847447 10.1007/s00401-009-0601-5 PMC2799635

[33] Fletcher JL, et al. Oligodendrogenesis and myelination regulate cortical development, plasticity and circuit function. Semin Cell Dev Biol. 2021;118:14–23. 33863642 10.1016/j.semcdb.2021.03.017

[34] Nave K-A, Ehrenreich H. Myelination and oligodendrocyte functions in psychiatric diseases. JAMA Psychiat. 2014;71(5):582–4. 10.1001/jamapsychiatry.2014.189 24671770

[35] Sturrock RR. Myelination of the mouse corpus callosum. Neuropathol Appl Neurobiol. 1980;6(6):415–20. 7453945 10.1111/j.1365-2990.1980.tb00219.x

[36] Mühlebner A, et al. Myelin pathology beyond white matter in tuberous sclerosis complex (TSC) cortical tubers. J Neuropathol Exp Neurol. 2020;79(10):1054–64. 32954437 10.1093/jnen/nlaa090 PMC7559237

[37] Peters JM, et al. White matter mean diffusivity correlates with myelination in tuberous sclerosis complex. Ann Clin Transl Neurol. 2019;6(7):1178–90. 31353853 10.1002/acn3.793 PMC6649396

[38] Scholl T, et al. Impaired oligodendroglial turnover is associated with myelin pathology in focal cortical dysplasia and tuberous sclerosis complex. Brain Pathol. 2017;27(6):770–80. 27750396 10.1111/bpa.12452 PMC5697648

[39] Ercan E, et al. Neuronal CTGF/CCN2 negatively regulates myelination in a mouse model of tuberous sclerosis complex. J Exp Med. 2017;214(3):681–97. 28183733 10.1084/jem.20160446 PMC5339668

[40] Gruber VE, et al. Impaired myelin production due to an intrinsic failure of oligodendrocytes in mTORpathies. Neuropathol Appl Neurobiol. 2021;47(6):812–25. 34173252 10.1111/nan.12744 PMC8518586

[41] Nadadhur AG, et al. Neuron-glia interactions increase neuronal phenotypes in tuberous sclerosis complex patient iPSC-derived models. Stem Cell Reports. 2019;12(1):42–56. 30581017 10.1016/j.stemcr.2018.11.019 PMC6335594

[42] Coppi E, et al. Role of adenosine in oligodendrocyte precursor maturation. Front Cell Neurosci. 2015;9:155. 25964740 10.3389/fncel.2015.00155 PMC4408841

[43] Chen, J.-F., C.-f. Lee, and Y. Chern, Chapter One - Adenosine Receptor Neurobiology: Overview, In: Mori A (Ed). International Review of Neurobiology, Academic Press. 2014;1–49. 10.1016/B978-0-12-801022-8.00001-5 25175959

[44] King AE, et al. Nucleoside transporters: from scavengers to novel therapeutic targets. Trends Pharmacol Sci. 2006;27(8):416–25. 16820221 10.1016/j.tips.2006.06.004

[45] Wu KC, et al. Preclinical evaluation of a novel molecule targeting nucleoside homeostasis to restore energy metabolism and cognitive function in Alzheimer’s disease. Pharmacol Res Perspect. 2025;13(5):e70176. 41046322 10.1002/prp2.70176 PMC12496225

[46] Lee CC, et al. Adenosine Augmentation Evoked by an ENT1 Inhibitor Improves Memory Impairment and Neuronal Plasticity in the APP/PS1 Mouse Model of Alzheimer’s disease. Mol Neurobiol. 2018;55(12):8936–52. 29616397 10.1007/s12035-018-1030-z

[47] Chang C-P, et al. Equilibrative nucleoside transporter 1 inhibition rescues energy dysfunction and pathology in a model of tauopathy. Acta Neuropathol Commun. 2021;9(1):112. 34158119 10.1186/s40478-021-01213-7 PMC8220833

[48] Chang CP, et al. Emerging roles of dysregulated adenosine homeostasis in brain disorders with a specific focus on neurodegenerative diseases. J Biomed Sci. 2021;28(1):70. 34635103 10.1186/s12929-021-00766-y PMC8507231

[49] Tordoff MG, et al. The maintenance diets of C57BL/6J and 129X1/SvJ mice influence their taste solution preferences: implications for large-scale phenotyping projects. J Nutr. 2002;132(8):2288–97. 12163677 10.1093/jn/132.8.2288 PMC2486364

[50] Schindelin J, et al. Fiji: an open-source platform for biological-image analysis. Nat Methods. 2012;9(7):676–82. 22743772 10.1038/nmeth.2019 PMC3855844

[51] Hsieh CC, et al. Detection of endophenotypes associated with neuropsychiatric deficiencies in a mouse model of tuberous sclerosis complex using diffusion tensor imaging. Brain Pathol. 2021;31(1):4–19. 32530070 10.1111/bpa.12870 PMC8018051

[52] Arshadi C, et al. SNT: a unifying toolbox for quantification of neuronal anatomy. Nat Methods. 2021;18(4):374–7. 33795878 10.1038/s41592-021-01105-7

[53] Hsieh CC, et al. Amelioration of the brain structural connectivity is accompanied with changes of gut microbiota in a tuberous sclerosis complex mouse model. Transl Psychiatry. 2024;14(1):68. 38296969 10.1038/s41398-024-02752-y PMC10830571

[54] Yeh F-C. DSI studio: an integrated tractography platform and fiber data hub for accelerating brain research. Nat Methods. 2025;22(8):1617–9. 40707713 10.1038/s41592-025-02762-8 PMC12394933

[55] Garyfallidis, E., et al., *Dipy, a library for the analysis of diffusion MRI data.* Frontiers in Neuroinformatics, 2014;8. 10.3389/fninf.2014.00008 PMC3931231 24600385

[56] Arulrajah S, et al. Magnetic resonance imaging and diffusion-weighted imaging of normal-appearing white matter in children and young adults with tuberous sclerosis complex. Neuroradiology. 2009;51(11):781–6. 19603155 10.1007/s00234-009-0563-2

[57] Lee SE, et al. Enhanced phosphorylation of S6 protein in mouse cortical layer V and subplate neurons. NeuroReport. 2020;31(10):762–9. 32453020 10.1097/WNR.0000000000001479

[58] Ricciardi S, et al. Reduced AKT/mTOR signaling and protein synthesis dysregulation in a Rett syndrome animal model. Hum Mol Genet. 2011;20(6):1182–96. 21212100 10.1093/hmg/ddq563

[59] Peters JM, et al. Longitudinal effects of Everolimus on white matter diffusion in tuberous sclerosis complex. Pediatr Neurol. 2019;90:24–30. 30424962 10.1016/j.pediatrneurol.2018.10.005 PMC6314307

[60] Tillema JM, et al. Everolimus alters white matter diffusion in tuberous sclerosis complex. Neurology. 2012;78(8):526–31. 22262746 10.1212/WNL.0b013e318247ca8d

[61] Baumer FM, et al. Corpus callosum white matter diffusivity reflects cumulative neurological comorbidity in tuberous sclerosis complex. Cereb Cortex. 2018;28(10):3665–72. 29939236 10.1093/cercor/bhx247 PMC6132277

[62] Makki MI, et al. Characteristics of abnormal diffusivity in normal-appearing white matter investigated with diffusion tensor MR imaging in tuberous sclerosis complex. AJNR Am J Neuroradiol. 2007;28(9):1662–7. 17893226 10.3174/ajnr.A0642 PMC8134198

[63] Dogan MS, et al. Brain diffusion tensor imaging in children with tuberous sclerosis. Diagn Interv Imaging. 2016;97(2):171–6. 25936891 10.1016/j.diii.2015.04.002

[64] Peters J, et al. Loss of white matter microstructural integrity is associated with adverse neurological outcome in tuberous sclerosis complex (S28.003). Neurology. 2012. 10.1212/wnl.78.1_meetingabstracts.s28.003. 22142677 10.1016/j.acra.2011.08.016 PMC3343770

[65] Jensen JH, et al. Diffusional kurtosis imaging: The quantification of non-gaussian water diffusion by means of magnetic resonance imaging. Magn Reson Med. 2005;53(6):1432–40. 15906300 10.1002/mrm.20508

[66] Lebrun-Julien F, et al. Balanced mTORC1 activity in oligodendrocytes is required for accurate CNS myelination. J Neurosci. 2014;34(25):8432–48. 24948799 10.1523/JNEUROSCI.1105-14.2014 PMC6608214

[67] Jiang M, et al. Regulation of PERK–eIF2α signalling by tuberous sclerosis complex-1 controls homoeostasis and survival of myelinating oligodendrocytes. Nat Commun. 2016;7(1):12185. 27416896 10.1038/ncomms12185 PMC4947172

[68] Carson RP, et al. Neuronal and glia abnormalities in Tsc1-deficient forebrain and partial rescue by rapamycin. Neurobiol Dis. 2012;45(1):369–80. 21907282 10.1016/j.nbd.2011.08.024 PMC3225598

[69] Meikle L, et al. Response of a neuronal model of tuberous sclerosis to mammalian target of rapamycin (mTOR) inhibitors: effects on mTORC1 and Akt signaling lead to improved survival and function. J Neurosci. 2008;28(21):5422–32. 18495876 10.1523/JNEUROSCI.0955-08.2008 PMC2633923

[70] Ozcan U, et al. Loss of the tuberous sclerosis complex tumor suppressors triggers the unfolded protein response to regulate insulin signaling and apoptosis. Mol Cell. 2008;29(5):541–51. 18342602 10.1016/j.molcel.2007.12.023 PMC2361721

[71] Figlia G, Gerber D, Suter U. Myelination and mTOR. Glia. 2018;66(4):693–707. 29210103 10.1002/glia.23273 PMC5836902

[72] Tang G, et al. Loss of mTOR-dependent macroautophagy causes autistic-like synaptic pruning deficits. Neuron. 2014;83(5):1131–43. 25155956 10.1016/j.neuron.2014.07.040 PMC4159743

[73] Yasin SA, et al. mTOR-dependent abnormalities in autophagy characterize human malformations of cortical development: evidence from focal cortical dysplasia and tuberous sclerosis. Acta Neuropathol. 2013;126(2):207–18. 23728790 10.1007/s00401-013-1135-4

[74] Di Nardo A, et al. Neuronal Tsc1/2 complex controls autophagy through AMPK-dependent regulation of ULK1. Hum Mol Genet. 2014;23(14):3865–74. 24599401 10.1093/hmg/ddu101 PMC4065158

[75] Goto J, et al. Regulable neural progenitor-specific Tsc1 loss yields giant cells with organellar dysfunction in a model of tuberous sclerosis complex. Proc Natl Acad Sci U S A. 2011;108(45):E1070–9. 22025691 10.1073/pnas.1106454108 PMC3214999

[76] Ebrahimi-Fakhari D, et al. Impaired Mitochondrial Dynamics and Mitophagy in Neuronal Models of Tuberous Sclerosis Complex. Cell Rep. 2016;17(4):1053–70. 27760312 10.1016/j.celrep.2016.09.054 PMC5078873

[77] Sade AN, Wiener G, Barak B. Intersection of mitochondrial dysfunction and myelination: An overlooked aspect in neurodevelopmental disorders. Neural Regen Res. 2026;21(2):659–60. 39995084 10.4103/NRR.NRR-D-24-01025 PMC12220700

[78] Cherchi F, Pugliese AM, Coppi E. Oligodendrocyte precursor cell maturation: role of adenosine receptors. Neural Regen Res. 2021;16(9):1686–92. 33510056 10.4103/1673-5374.306058 PMC8328763

[79] Shen H.-Y. et al. Adenosine Actions on Oligodendroglia and Myelination in Autism Spectrum Disorder. Frontiers in Cellular Neuroscience, 2018; 12. 10.3389/fncel.2018.00482 PMC6292987 30581380

[80] Stockwell J, Jakova E, Cayabyab FS. Adenosine A1 and A2A receptors in the brain: current research and their role in neurodegeneration. Molecules. 2017. 10.3390/molecules22040676. 28441750 10.3390/molecules22040676 PMC6154612

[81] Wei CJ, Li W, Chen JF. Normal and abnormal functions of adenosine receptors in the central nervous system revealed by genetic knockout studies. Biochim Biophys Acta. 2011;1808(5):1358–79. 21185258 10.1016/j.bbamem.2010.12.018

[82] Dunwiddie TV, Masino SA. The role and regulation of adenosine in the central nervous system. Annu Rev Neurosci. 2001;24:31–55. 11283304 10.1146/annurev.neuro.24.1.31

[83] de Faria Jr O, et al. Activity-dependent central nervous system myelination throughout life. J Neurochem. 2019;148(4):447–61. 30225984 10.1111/jnc.14592 PMC6587454

[84] Gibson EM, et al. Neuronal activity promotes oligodendrogenesis and adaptive myelination in the mammalian brain. Science. 2014;344(6183):1252304. 24727982 10.1126/science.1252304 PMC4096908

[85] Foutch K, et al. Adolescent seizure impacts oligodendrocyte maturation, neuronal-glial circuit Formation, and myelination in the mammalian forebrain. Neuroscience. 2025;564:144–59. 39571961 10.1016/j.neuroscience.2024.11.050

