# Supplementary figures and images for: ENT1 inhibitor J4 restores cognitive function and white-matter integrity in a mouse model of tuberous sclerosis complex
Source: J Biomed Sci. 2026 Jun 17;33:63. doi: 10.1186/s12929-026-01269-4 (PMC13274077; doi:10.1186/s12929-026-01269-4)

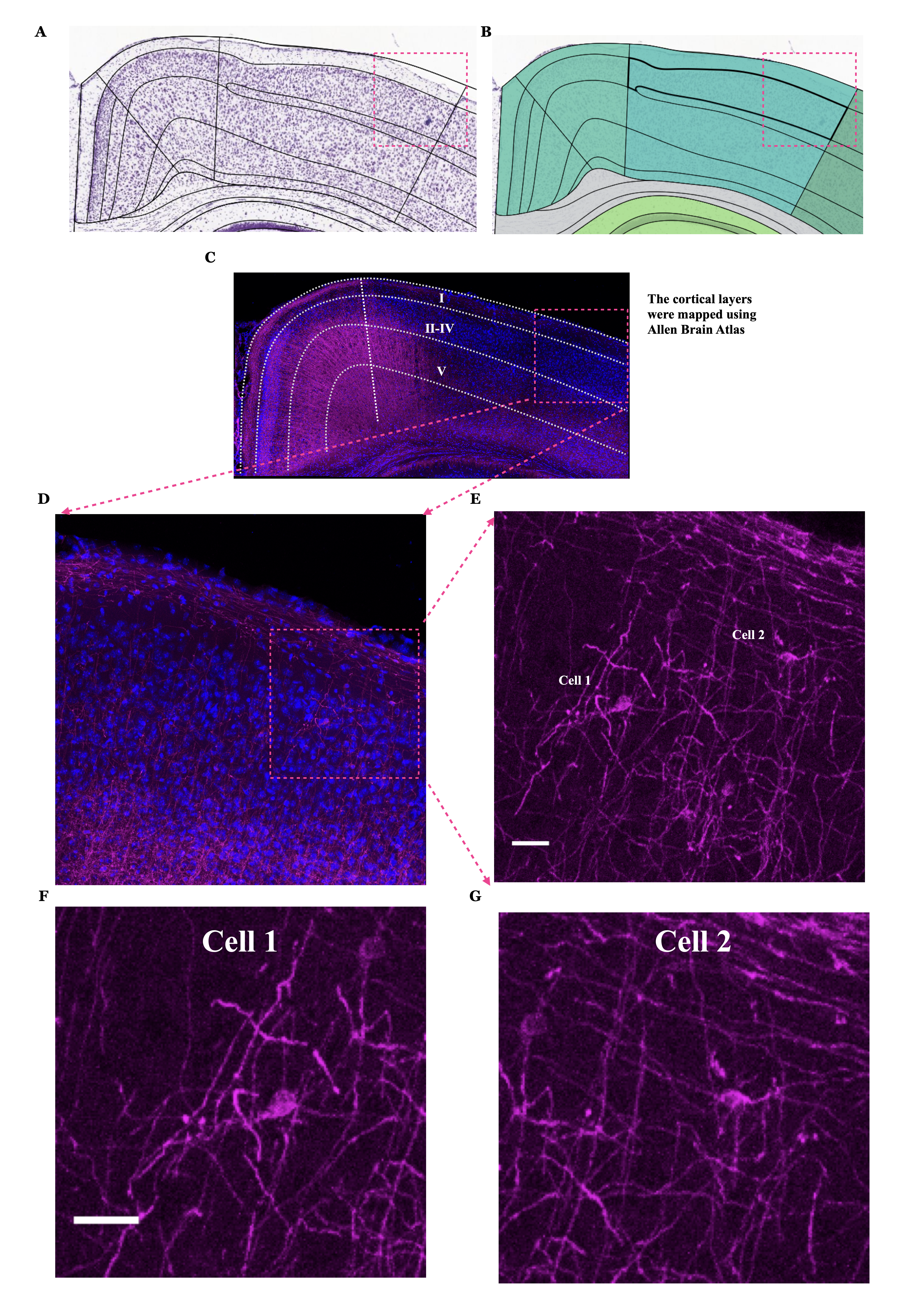

Supplement: Supplementary file 1 — Supplementary Material 1. [file 12929_2026_1269_MOESM1_ESM.tiff]

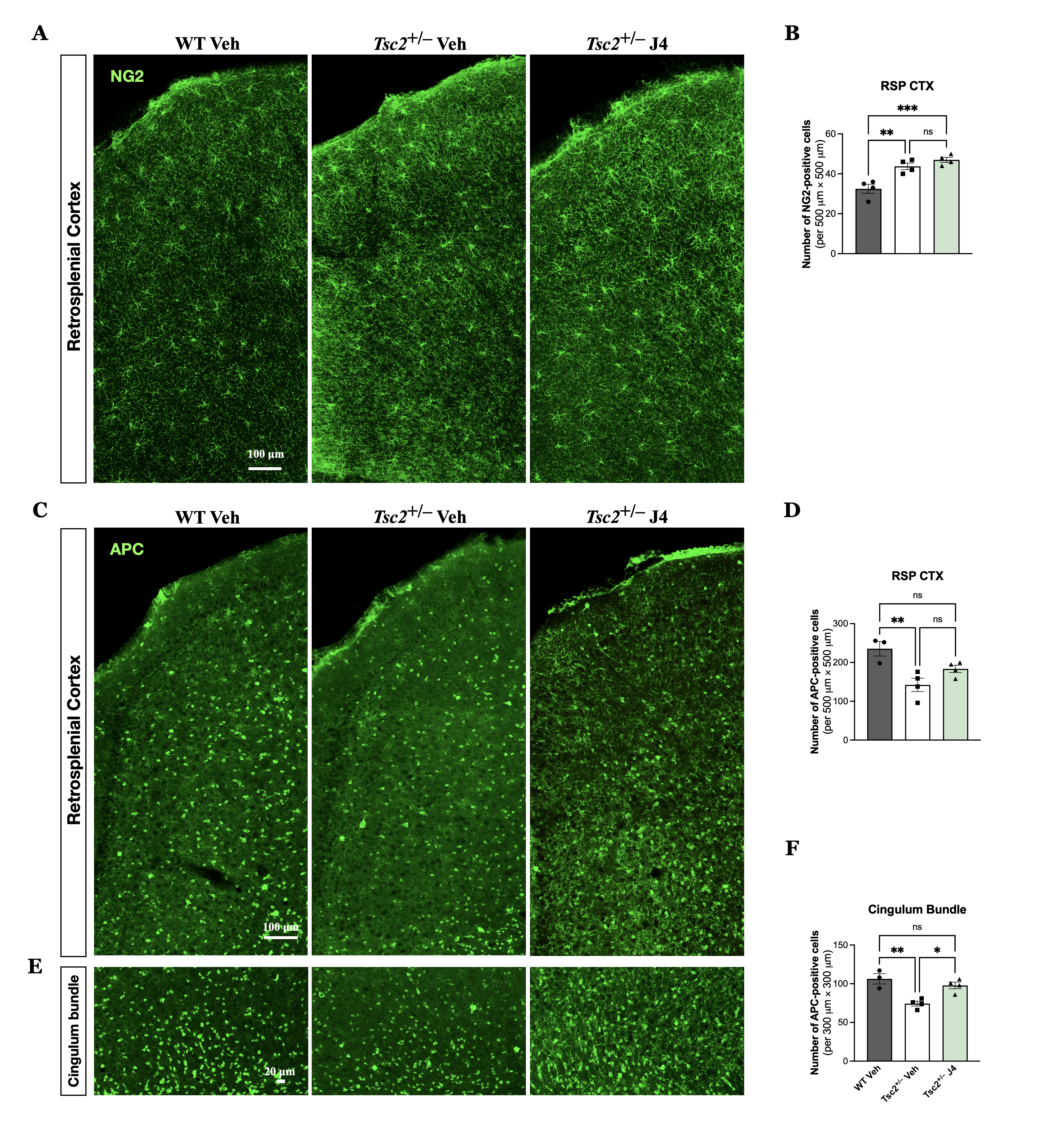

Supplement: Supplementary file 2 — Supplementary Material 2. [file 12929_2026_1269_MOESM2_ESM.tiff]
