# Supplementary material for: ENT1 inhibitor J4 restores cognitive function and white-matter integrity in a mouse model of tuberous sclerosis complex
Source: J Biomed Sci. 2026 Jun 17;33:63. doi: 10.1186/s12929-026-01269-4 (PMC13274077; doi:10.1186/s12929-026-01269-4)
Supplement: Supplementary file 4 — Supplementary Material 4. [file 12929_2026_1269_MOESM4_ESM.docx]

**Supporting information**

**Supplementary Fig. 1**. Atlas-based definition of cortical layers. **(A–B)** Representative coronal section of the mouse brain and corresponding schematic mapping based on the Allen Brain Atlas. The retrosplenial (RSP), parietal, and somatosensory (SS) cortices and cortical layer boundaries are identified according to atlas coordinates. **(C)** Overlay of cortical layer boundaries (layers II–IV and V) onto immunofluorescence images of the RSP. Layer assignment was performed by aligning anatomical representations (based on the cell density and size) with the reference atlas to ensure consistent region-of-interest (ROI) definition across samples. **(D–E)** Higher-magnification views of the RSP region used for analysis, indicating the areas from which oligodendrocytes were selected for morphological reconstruction. **(F–G)** Representative examples of CNPase-positive oligodendrocytes (Cell 1 and Cell 2) within the defined cortical layers. Cells were selected based on clearly identifiable cell bodies and processes within the mapped ROI.

**Supplementary Fig. 2. J4 treatment increased APC-positive cells in the cingulum bundles, but not retrosplenial cortex**

**(A)** Representative immunofluorescence images from WT, *Tsc2^+/–^* Veh, and *Tsc2^+/–^* J4 groups showing staining of NG2, a marker of oligodendrocyte progenitor cells, in retrosplenial cortex. **(B)** Quantitative results showing the number of NG2-positive cells in each group. **(C)** Representative immunofluorescence images from WT, *Tsc2^+/–^* Veh, and *Tsc2^+/–^* J4 groups showing staining of APC, a marker of immature and mature oligodendrocytes, in retrosplenial cortex. **(D)** Quantitative results showing the number of APC-positive cells in each group. **(E)** Representative immunofluorescence images showing staining of APC in the cingulum bundle. **(F)** Quantitative results showing the number of APC-positive cells in the cingulum bundle for each group.

**Supplementary Fig. 3. Laminar distribution of pS6 expression in the mouse cortex.**

Representative immunofluorescence images showing co-staining of phosphorylated S6 (pS6, green) and Ctip2 (red), a marker of cortical layer V neurons, in the **(A)** Somatosensory cortex and **(B)** the retrosplenial cortex. Nuclei are counterstained with DAPI (blue). The merged images demonstrate that pS6 signal is predominantly localized in Ctip2-positive neurons, indicating enrichment of pS6 expression in layer V. Yellow arrows indicate regions with elevated pS6 signal in layers II-IV in *Tsc2^+/–^* Veh mice, while red arrows indicate increased pS6 signal in layer V. Comparable anatomical regions are indicated with white arrows in WT Veh. Scale bars: 100 μm.
